# Identification of flux checkpoints in a metabolic pathway through white-box, grey-box and black-box modeling approaches

**DOI:** 10.1038/s41598-020-70295-5

**Published:** 2020-08-10

**Authors:** Ophélie Lo-Thong, Philippe Charton, Xavier F. Cadet, Brigitte Grondin-Perez, Emma Saavedra, Cédric Damour, Frédéric Cadet

**Affiliations:** 1 University of Paris, UMR_S1134, BIGR, Inserm, 75015 Paris, France; 2 DSIMB, UMR_S1134, BIGR, Inserm, Laboratory of Excellence GR-Ex, Faculty of Sciences and Technology, University of La Reunion, 97715 Saint-Denis, France; 3 PEACCEL, Artificial Intelligence Department, 6 square Albin Cachot, box 42, 75013 Paris, France; 4 LE2P, Laboratory of Energy, Electronics and Processes EA 4079, Faculty of Sciences and Technology, University of La Reunion, 97444 St Denis cedex, France; 5 Departamento de Bioquímica, Instituto Nacional de Cardiología Ignacio Chávez, 14080 Mexico City, Mexico

**Keywords:** Computational models, Machine learning

## Abstract

Metabolic pathway modeling plays an increasing role in drug design by allowing better understanding of the underlying regulation and controlling networks in the metabolism of living organisms. However, despite rapid progress in this area, pathway modeling can become a real nightmare for researchers, notably when few experimental data are available or when the pathway is highly complex. Here, three different approaches were developed to model the second part of glycolysis of *E. histolytica* as an application example, and have succeeded in predicting the final pathway flux: one including detailed kinetic information (white-box), another with an added adjustment term (grey-box) and the last one using an artificial neural network method (black-box). Afterwards, each model was used for metabolic control analysis and flux control coefficient determination. The first two enzymes of this pathway are identified as the key enzymes playing a role in flux control. This study revealed the significance of the three methods for building suitable models adjusted to the available data in the field of metabolic pathway modeling, and could be useful to biologists and modelers.

## Introduction

*Entamoeba histolytica* is a protozoan parasite responsible for the development of amoebiasis in humans. This disease is a worldwide public health problem that causes over 100 000 deaths per year^1^. Indeed, a recent report estimates the prevalence of *E. histolytica* infection at 42% in Mexico, 41% in China and 34% in South Africa^2^. So far, no vaccine exists to prevent the infection, but patients who suffer from amoebiasis can be treated with different drugs such as metronidazole or tinidazole. However, intolerances to these treatments and potential appearance of drug resistance^2–5^ reveal the urgency of the situation and the need to find new therapies. Previous studies have focused on the identification of new drug targets in *E. histolytica* glycolysis^6–8^, since the parasite depends completely on glycolysis to produce ATP^9^.

While drug research and development is time consuming and expensive, the use of computational approaches might help to speed up the process. Lately, the combination of in vitro reconstitution and in silico modeling of the glycolysis pathway in *E. histolytica* highlighted the possibility of using modeling for predicting flux and metabolite concentrations under given conditions^7^ and for appraising the effect of the addition of alternative routes^8^. Pathway modeling can be done through many statistical or knowledge driven approaches^10^. The first one only uses experimental data to understand relationships between biological variables, whereas the second uses pathway information (metabolic reactions, thermodynamic and kinetic parameters) to design complete detailed metabolic pathway reconstructions. Artificial Neural Network (ANN) can be classified among the data-driven approaches and is based on the creation of a network whose structure and functioning are similar to those of a biological neural network^11^. Traditionally, this method is employed to identify new biomarkers of diseases such as cancer^11^ or to predict the bioavailability of drugs in patients^12,13^.

**Figure 1:**
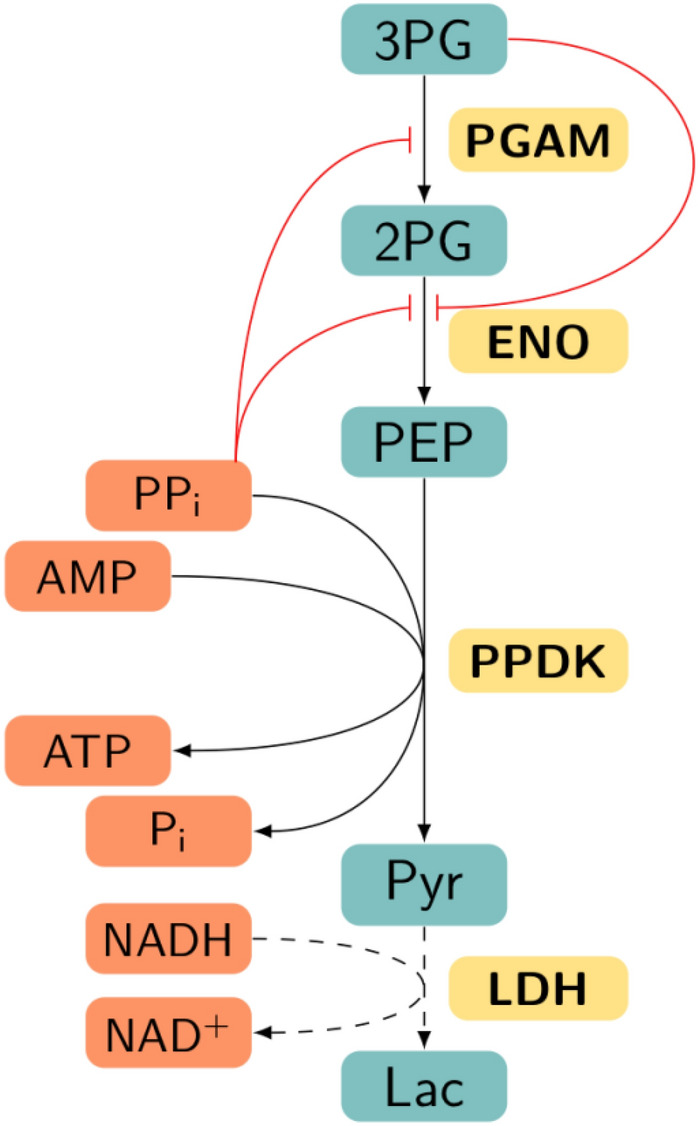
Second part of *E. histolytica* glycolysis pathway. The pathway is formed by 3-phosphoglycerate mutase (PGAM), enolase (ENO) and pyruvate phosphate dikinase (PPDK). Reduction of pyruvate to L-lactate (Lac) consuming NADH (dashed lines) is not part of the parasite pathway, but lactate dehydrogenase (LDH) was used in the in vitro reconstituted pathway in order to experimentally follow the final flux and establish a quasi steady-state to Lac ^8^. Metabolite action in enzyme inhibition is represented in red. *3PG* 3-phosphoglycerate; *2PG* 2-phosphoglycerate; *PEP* phosphoenolpyruvate; *Pyr* pyruvate.

The recent model of *E. histolytica* glycolysis applies a knowledge-based method called metabolic network to each part of the pathway: the first part from glucose to dihydroxyacetone phosphate and the second part (Fig. 1) from 3-phosphoglycerate (3PG) to pyruvate (Pyr)^8^. Interestingly, these studies found that 3-phoshoglycerate mutase (PGAM) was the main controlling factor in the second part of glycolysis, whereas pyruvate phosphate dikinase (PPDK) exerted the lowest flux control. This result comes in conflict with previous research^6^, which identified PGAM and PPDK as important flux control steps of amoebal glycolysis. This difference is explained by the use of inappropriate enzyme proportions in the in vitro reconstitution experiments, not identical to those determined in amoebas, in the first study. Moreover, here our study is based on the experimental results of Moreno-Sanchez^8^.

It should be noted that obtaining a solid knowledge-based model relies, as the name suggests, upon an advanced understanding of the cell system, including physiological metabolite concentrations and enzyme activities, kinetic parameters and the type of mechanism involved, as well as thermodynamic constants of the pathway reactions. However, this knowledge is often not available in the literature or is highly complex to model, as seen with the kinetic mechanism of PPDK^8,14^.

In the present study, our objective is to contribute to overcome the lack of knowledge and the complexity of kinetic modeling (white-box modeling), by testing two new modeling approaches: a data-driven approach (black-box modeling) which uses ANN model, and a hybrid-based approach (grey-box modeling) which uses a traditional kinetic-based model with an added adjustment term. For this purpose, these three modeling approaches are applied to an experimental example: the second part of *E. histolytica* glycolysis, using the experimental results previously published by Moreno-Sanchez et al.^8^

Our analysis shows that the different models predict correctly the final flux values in the second part of *E. histolytica* glycolysis pathway. The ANN model presents great predictive and generalization abilities; however, its complexity, through high Akaike Information Criterion value (AIC), ranks it among the less satisfactory models. The COPASI models provide satisfactory predicted fluxes, as well as the ANN model, with a marked preference for the grey-box approach. Subsequently, the flux control coefficients of the enzymes (${C}_{E}^{J}$) are calculated and allow the identification of the key enzymes involved in flux control^15–17^. Taken together, these models enable the construction of the pathway from experimental data and the determination of the main controlling enzymes in the system, revealing the relevance of both the traditional white-box approach and the novel grey- and black-box approach. Such approaches could be extended to further biological pathway modeling, as they provide models adapted to various backgrounds.

## Materials and methods

### Second part of glycolysis experimental data

Experimental data of PGAM, ENO and PPDK activities and pathway flux (*J*_*obs*_) are obtained from plots of a previous study^8^. The free online software WebPlotDigitizer (Version 4.1, https://automeris.io/WebPlotDigitizer/) is used to extract data from plots. These data are available in Tables S1 and S2.

### Artificial neural networks (ANNs)

ANNs functioning mimics that of biological neurons, the networks consist of many layers allowing input reception and processing and output delivery. This technique can be used for solving classification or regression problems^18^. To build the second part of glycolysis in ANNs, different types of software are employed: RStudio (Version 1.1.456), an open-source integrated development environment for R^19^ and two packages: NeuralNet (Version 1.44.2) and Nnet (Version 7.3–12)^20,21^.

### Complex pathway SImulator (COPASI) metabolic networks

A first metabolic network of the studied pathway was kindly provided by the authors of a previous study^8^. This model is developed on GEPASI^22^, an old software for metabolic pathway modeling, replaced by COPASI since 2002.

The second part of the glycolysis is also modeled by using the open source software called COPASI (Version 4.24)^23^. This software is used for metabolic network design, analysis and optimization. The resulting metabolic networks are based on the use of enzyme properties (kinetic parameters and mechanism-based rate equations).

### Ethics approval and consent to participate

Not applicable.

## Methodology

### Black- white- and grey-box approach procedure

**Figure 2:**
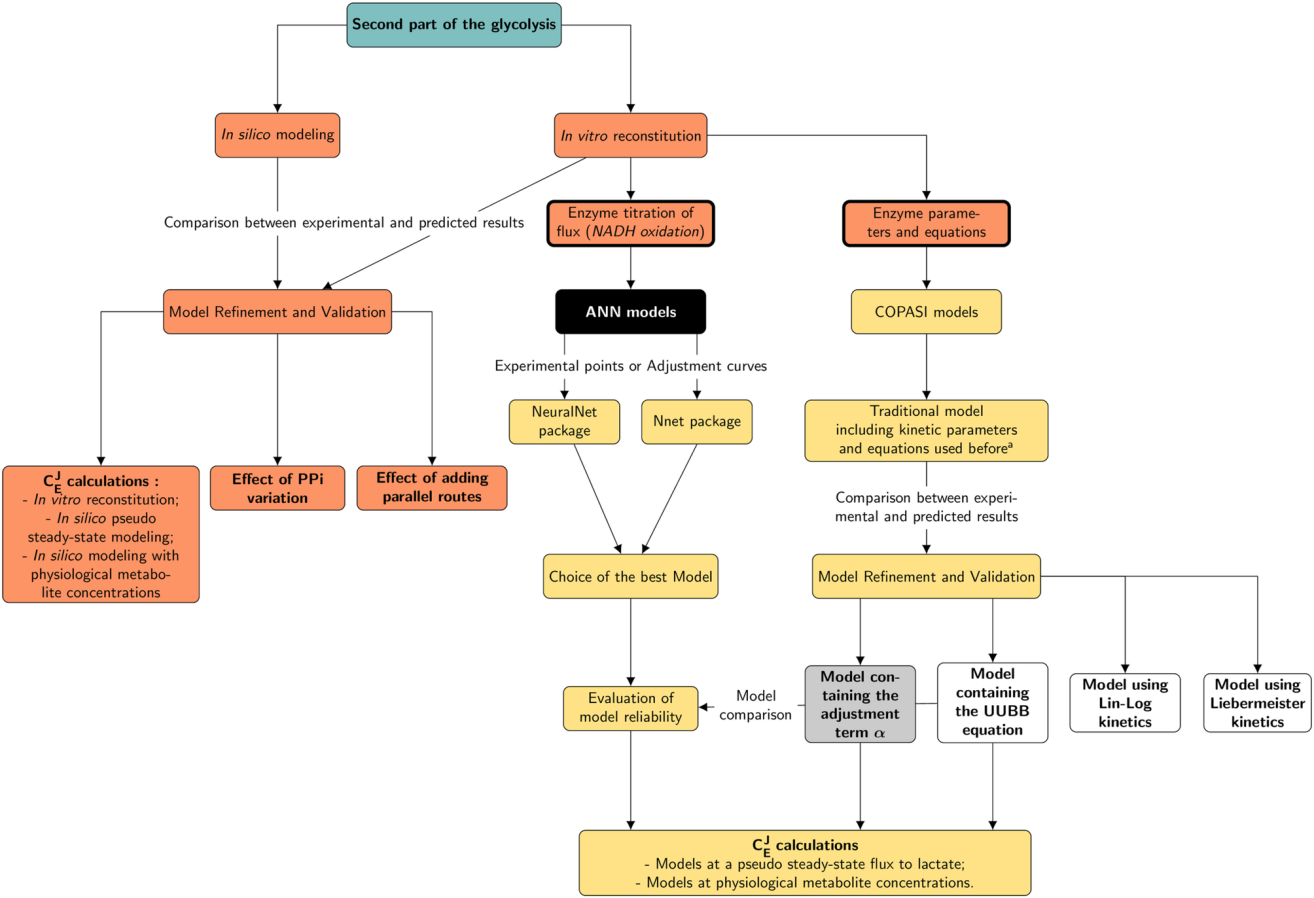
Study workflow. Moreno-Sanchez et al. methodology^8^ is represented in orange, whereas the methodology proposed here is represented in yellow. Boxes with a thick line indicate the experimental data used in this study; left box: the flux mentioned here refers to pathway flux titration by changing enzyme activities. The last boxes are the techniques used for a better understanding of the metabolic pathway. The five final models designed in this work are colored in black, white or grey. ^a^See “*Complex Pathway SImulator (COPASI) metabolic networks*” part.

To conduct the present study, a specific methodology, different from that envisaged in the original article^8^, is defined (Fig. 2). In the first case of the black-box approach, ANN models are built with the experimental data concerning the relationship of pathway flux *versus* enzyme activity in the pathway in vitro reconstruction. Then, in the second and third case of the white- and grey-box approach, metabolic networks are built with enzyme parameters measured experimentally, and rate equations^24^ according to the type of kinetic mechanism described for each enzyme. Once the models are designed, a comparison of their final flux and product concentrations is made. Also, for each approach, two different models are designed: one reaching a pseudo-steady-state flux through lactate and another at physiological metabolite concentrations. Subsequently, calculations of flux control coefficient for each of these models are made, allowing the determination of the main flux controlling enzyme.

### Black-box approach

#### Artificial neural networks (ANNs) design

**Figure 3:**
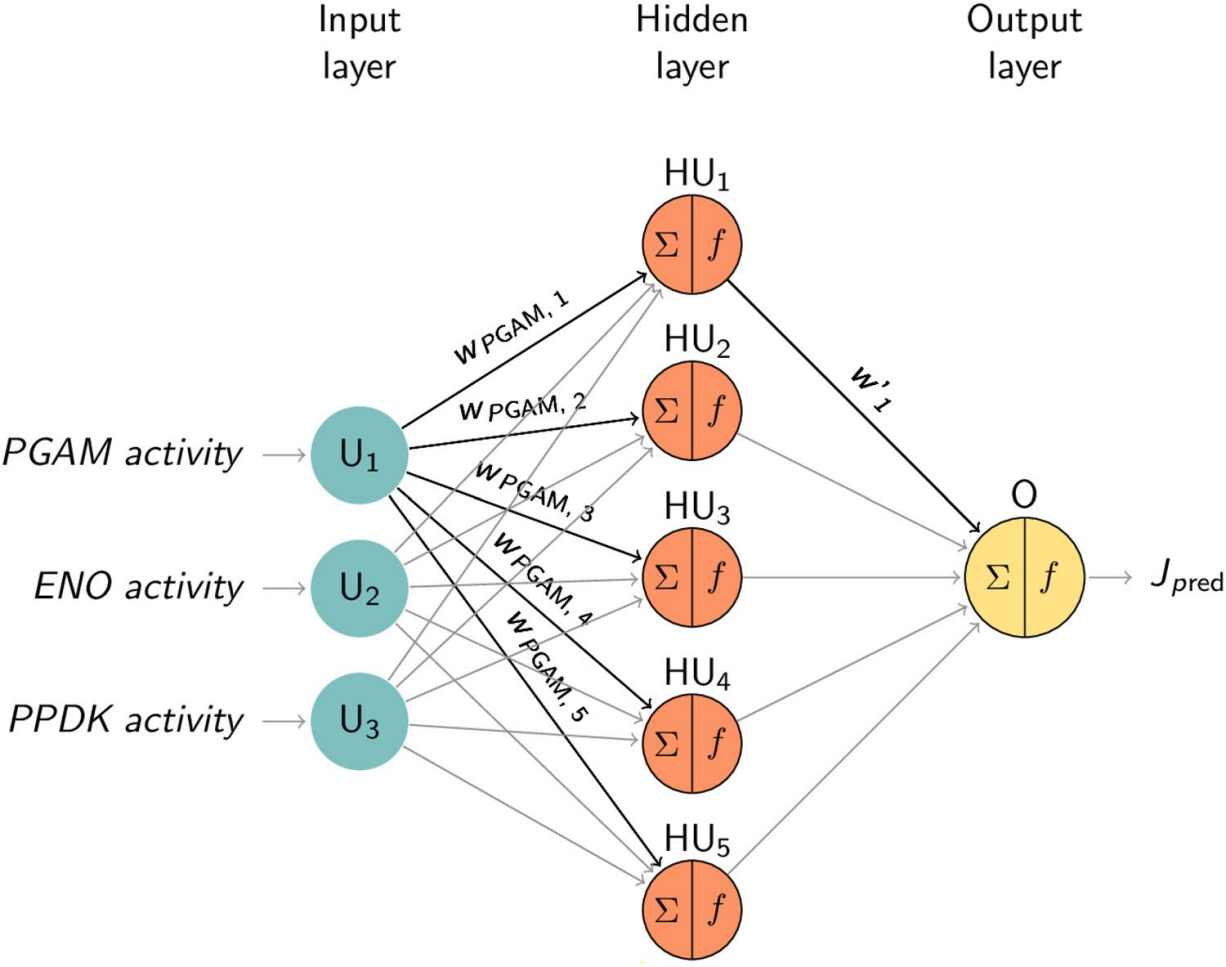
Structure of the ANN models. Each node represents an artificial neuron or unit. U_i_, HU_j_ and O are, respectively, the input unit, the hidden unit and the output unit of the different layers; *w*_*i*_ and *w’*_*j*_ are the weights associated with each connection of the network between the input and the hidden layer for the first, and between the hidden and the output layer for the second. Only weights for the first unit (associated with PGAM) of the layers are labelled. ∑ constitutes the weighted sum of the input and *f* constitutes the activation function applied in the unit.

Typical feed-forward networks are designed and consist of three layers of neurons: an input layer, a single hidden layer and an output layer (Fig. 3). Input data are connected to the neurons and weights (*w*_*i*_ and *w’*_*j*_) are assigned to each connection. When input data are processed by the neuron, the latter computes the weighted sum of its inputs, then applies an activation function (*f*). The activation function makes it possible to convert input into output and decides whether the neuron is activated or not. There are several activation functions, including the non-linear activation functions: logistic (log) and hyperbolic tangent (tanh). If the resulting output is higher than the set threshold, the neuron is considered as being activated, otherwise not. Lastly, the hidden layer leads to the final output result, displayed in the output layer.

Optimization of ANNs is ensured through the back-propagation method^25^ in the NeuralNet package and the Broyden-Fletcher-Goldfarb-Shanno (BFGS) method^26^ in the Nnet package. For detailed information on ANN functioning, see^27^. In the ANN models, the inputs are the activities of each enzyme (PGAM, ENO and PPDK) used in the in vitro experiment (Table S1,^8^), and the output is the predicted pathway flux (J_pred_). Also, each weight in the ANN is assigned automatically by RStudio. Given the small amount of experimental data (Table S1), ANN models are built with a training set made up of the complete Tables S1 or S2 datasets (the data from the experimental dots or data from the fitting curves, respectively), then optimized through a Leave-One-Out cross validation (LOOcv) procedure. Then, since we needed a separate test set to prevent overfitting, the models are evaluated on a different test set generated by the grey-box COPASI model (Table S3).

#### ANN selection and performance evaluation

The number of artificial neurons (or units) in the hidden layer is selected based on:

- the root-mean-square error (RMSE):
  1 $$RMSE = \sqrt{\frac{1}{n}\sum_{i=1}^{n}{({\widehat{Y_{i}}}-{Y}_{i})}^{2}}$$
  with ${Y}_{i}$ and ${\widehat{Y_{i}}}$ respectively the observed and predicted values, n the total number of values, and i = 1, 2…*n*;
- the mean absolute error (MAE) calculations:
  2 $$MAE = \frac{1}{n}\sum_{i=1}^{n}\left|{Y}_{i}-{\widehat{Y_{i}}}\right|$$
  with |…| symbolizing the absolute value;
- and a specific equation estimating a range of numbers of HUs^28,29^:
  3 $${N}_{h} =\frac{{N}_{s}}{\alpha *({N}_{i}+{N}_{o})}$$

with N_h_ the number of HUs, N_s_ the number of samples in the training data, N_i_ the number of input units, N_o_ the number of output units and $\alpha$ an arbitrary scaling factor, usually 2–10.

RMSE and MAE are statistical metrics commonly used to evaluate the model performance^30–33^.

### White-box approach

#### Complex Pathway SImulator (COPASI) metabolic network design

The metabolic networks built in this study use the enzyme properties (kinetic parameters and kinetic rate equations), which are summarized in Tables 1, 2, and metabolite concentrations defined in Table 3. Furthermore, several models are built using either *V*_*max*_ or *k*_*cat*_ and E and pseudo-steady state metabolite concentrations or physiological metabolite concentrations. All simulations are carried out during the first hour, as was done in the experimental procedure^8^.

**Table 1:** Kinetic parameters of the enzymes in the second part of the glycolysis.

Enzyme	*K*_*m*_^a^	*K*_*i*_^a^	*K*_*eq*_^a^	*V*_*max*_^a^	*k*_*cat*_^b^	E^c^
PGAM	473 (3PG) 106 (2PG)	173 (PPi)		*V*_*f*_ = 75 *V*_*r*_ = 67.24	*k*_*cat_f*_ = 3,420 *k*_*cat_r*_ = 3,066.14	2.19*10^–2^
ENO	86.4 (2PG) 102 (PEP)	137 (PPi) 610 (3PG)		*V*_*f*_ = 328.5 *V*_*r*_ = 66.61	*k*_*cat_f*_ = 8,820 *k*_*cat_r*_ = 1,788.43	3.72*10^–2^
PPDK	30 (PEP) 2 (AMP) 91 (PPi) 221 (Pyr) 597 (ATP) 1,342 (Pi)		0.73	*V*_*f*_= 196.5 *V*_*r*_ = 12.28	*k*_*cat_f*_ = 5,220 *k*_*cat_r*_ = 326.22	3.76*10^–2^

Michaelis constants (*K*_*m*_) and inhibitor constants (*K*_*i*_) are in µM, maximum rates of the forward and reverse reactions (*V*_*f*_ and *V*_*r*_) in mU, enzyme amounts (E) in nmol and *k*_*cat*_ of the forward and reverse reactions (*k*_*cat_f*_ and *k*_*cat_r*_) in min^−1^. *K*_*eq*_ is the equilibrium constant of the reaction.

^a^Data taken from a previous study^8^ and *V*_*r*_ were calculated from enzyme proportions^7^.

^b^Data taken from a previous study^6^ and *k*_*cat_r*_ were calculated from *V*_*r*_ and E.

^c^E were calculated from *V*_*f*_ and *k*_*cat_f*_ by using the equation: $E = \frac{{V}_{f}}{{k}_{cat\_f}}$.

**Table 2:** Kinetic equations of the enzymes in the second part of the glycolysis.

Enzyme	Kinetic equations^a^
PGAM	$v=\frac{{V}_{f}\frac{[3PG]}{{K}_{m3PG}}-{V}_{r}\frac{[2PG]}{{K}_{m2PG}}}{1+\frac{[3PG]}{{K}_{m3PG}}+\frac{[2PG]}{{K}_{m2PG}}+\frac{[{PP}_{i}]}{{K}_{i{PP}_{i}}}}$
ENO	$v=\frac{{V}_{f}\frac{[2PG]}{{K}_{m2PG}}-{V}_{r}\frac{[PEP]}{{K}_{mPEP}}}{1+\frac{[2PG]}{{K}_{m2PG}}+\frac{[PEP]}{{K}_{mPEP}}+\frac{[{PP}_{i}]}{{K}_{i{PP}_{i}}}+\frac{[3PG]}{{K}_{i3PG}}}$
PPDK^b^	$v=\frac{{V}_{f}\left(ABC-\frac{PQR}{{K}_{eq}}\right)}{{K}_{mA}B+{K}_{mB}A+{K}_{mC}B+{K}_{mB}C+\frac{{V}_{f}{K}_{mQ}P}{{V}_{r}{K}_{eq}}+\frac{{V}_{f}{K}_{mP}Q}{{V}_{r}{K}_{eq}}+\frac{{V}_{f}{K}_{mQ}R}{{V}_{r}{K}_{eq}}+\frac{{V}_{f}{K}_{mR}Q}{{V}_{r}{K}_{eq}}+ABC+\frac{{V}_{f}PQR}{{V}_{r}{K}_{eq}}}$

^a^In models using *k*_*cat*_ and E, *V*_*f*_ were replaced by ${k}_{cat\_f}\cdot E$ and *V*_*r*_ were replaced by ${k}_{cat\_r}\cdot E$.

^b^A, B and C and *K*_*mA*_, *K*_*mB*_ and *K*_*mC*_ are respectively the concentrations and *K*_*m*_ of the substrates PEP, AMP and PP_i_; P, Q and R and *K*_*mP*_, *K*_*mQ*_ and *K*_*mR*_ are the concentrations and *K*_*m*_ of the products Pyr, ATP, P_i_.

**Table 3:** Metabolite concentrations used in the models.

Metabolite	Pseudo-steady state concentrations (in µM)^a^	Physiological concentrations (in µM)^b^
3PG	4,000	400
AMP	200	1,600
PP_i_	1,700	450
ATP	3,000	5,000
P_i_	10,000	5,400

^a^See Tables 1, 2 of Ref.^8^.

^b^See Table 3 of Ref.^8^.

As in the previous study, for establishing a quasi steady-state and calculating the flux control coefficients during modeling, a last reaction is added: Lac formation from Pyr (Fig. 1). The kinetic equation of LDH is $k \times [Pyr]$, with the rate constant $k=2,000$ min^−1^, and the Lac concentration is fixed at 300 µM.

### Metabolic network refinement and validation

To enhance the COPASI model predictions, changes to their contents are carried out. First of all, the PPDK kinetic equation is modified and a more accurate one describing the full rate equation is used, the Uni Uni Bi Bi Ping-Pong (UUBB) mechanism (Eq. 4) as previously determined^14^:

4 $${v}_{2}=\frac{{V}_{f}{V}_{r}\left(ABC-\frac{PQR}{{K}_{eq}}\right)}{D}$$

with the denominator $\text{D} = {V}_{r}{K}_{iB}{K}_{C}\text{A}+{V}_{r}{K}_{C}\text{AB}+{V}_{r}{K}_{B}\text{AC}+\frac{{V}_{f}}{{K}_{eq}}{K}_{iR}{K}_{Q}\text{P}+\frac{{V}_{f}}{{K}_{eq}}{K}_{R}\text{PQ}+{V}_{r}{K}_{iB}\frac{{K}_{C}}{{K}_{iQ}}\text{AQ}+\frac{{V}_{f}}{{K}_{eq}}{K}_{Q}\text{PR}+\frac{{V}_{f}}{{K}_{eq}}{K}_{P}\text{QR}+\frac{{V}_{f}}{{K}_{eq}}{K}_{Q}\text{PR}+ {V}_{r}{K}_{A}\text{BC}+\frac{{V}_{f}}{{K}_{eq}}\frac{{K}_{iR}{K}_{Q}}{{K}_{iC}}\text{CP}+{V}_{r}\frac{{K}_{c}}{{K}_{iQ}}\text{ABQ}+\frac{{V}_{f}}{{K}_{eq}}\frac{{K}_{R}}{{K}_{iA}}\text{APQ}+\frac{{V}_{f}}{{K}_{eq}}\frac{{K}_{P}}{{K}_{CB}}\text{BQR}+{V}_{r}\frac{{K}_{A}}{{K}_{iP}}\text{ACP}+{V}_{r}\frac{{K}_{A}}{{K}_{iR}}\text{BCR}+\frac{{V}_{f}}{{K}_{eq}}\frac{{K}_{P}}{{K}_{iB}}\text{BQR}+{V}_{r}\frac{{K}_{C}}{{K}_{iQ}{K}_{iiC}}\text{ABCQ}+\frac{{V}_{f}}{{K}_{eq}}\frac{{K}_{Q}}{{K}_{iC}}\text{CPR}+\frac{{V}_{f}}{{K}_{eq}}\frac{{K}_{Q}}{{K}_{iC}{K}_{iiC}}\text{CPR}+{V}_{r}\frac{{K}_{A}}{{K}_{iR}{K}_{iiC}}\text{BCQR}+{V}_{r}\text{ABC}+\frac{{V}_{f}}{{K}_{eq}}\text{PQR}+\frac{{V}_{f}}{{K}_{eq}}\frac{{K}_{iR}{K}_{Q}}{{K}_{iA}}\text{AP};$ A, B and C and P, Q and R are respectively the concentrations of the substrates PEP, AMP and PP_i_ and of the products Pyr, P_i_ and ATP of PPDK reaction; *K* is the Michaelis constant; *K*_*i*_ and *K*_*ii*_ are respectively the dissociation constant of the substrate or product and the inhibitor constant that affects the intercept (1/*V*_*max*_). The experimental and fitted constants are listed in Table 4.

**Table 4:** *K*_*i*_, *K*_*ii*_ and *K*_*PPi_AMP*_ used in the UUBB equation.

Constant	Value (in µM)
*K*_*ii_Pi*_	7,200
*K*_*i_Pyr*_	2,300
*K*_*i_Pi*_	23,000
*K*_*i_ATP*_	140
*K*_*ii_PPi*_^a^	1,000
*K*_*i_PEP*_^a^	1,000
*K*_*i_AMP*_^a^	1,000
*K*_*PPi_AMP*_^a^	1,000
*K*_*i_PPi*_^a^	1,000

^a^Fixed at an arbitrary value.

Also, the estimation of kinetic parameters is made with COPASI Parameter Estimation task. With this task, a range of parameters is tested by COPASI, which predicts the final flux or the product concentrations and compares them to the experimental data. The process relies on the minimization of the cost function (5), i.e. the minimization of the error between the experimental values and the corresponding predicted values.

5 $$E\left(P\right)= \sum_{i,j}{\omega }_{j}.{({x}_{i,j}-{y}_{i,j}\left(P\right))}^{2}$$

with E the calculated error, P the tested parameter, ${\omega }_{j}$ is the calculated weight for each experimental data column, ${x}_{i,j}$ a point in the dataset and ${y}_{i,j}\left(P\right)$ the corresponding predicted value; i and j are the rows and columns in the experimental dataset. The weight calculation method was the mean square: ${\omega }_{j}=\frac{1}{\overline{{{x}^{2}}_{j}}}$, with $\overline{{{x}^{2}}_{j}}$ the mean of squared data from one column. The software provides a list of optimization methods, to find optimized values for the estimated parameters (https://copasi.org/Support/User_Manual/Methods/Optimization_Methods/).

Again, two types of estimations are carried out:

- one estimating one or several parameters with one target value and
- the other estimating one or several parameters with many target values.

The models obtained constitute the white-box approach, with known enzymatic parameters and equations.

### Grey-box approach

In the specific case of the grey-box approach, to improve the COPASI model predictions, the kinetic equation of PPDK is changed to a ter-reactant reversible equations^8^ which was modified as follows (6):

6 $${v}_{1}=\frac{{V}_{f}\left(ABC-\frac{PQR}{{K}_{eq}}\right)}{{K}_{mA}B+{K}_{mB}A+{K}_{mC}B+{K}_{mB}C+\frac{{V}_{f}{K}_{mQ}P}{{V}_{r}{K}_{eq}}+\frac{{V}_{f}{K}_{mP}Q}{{V}_{r}{K}_{eq}}+\frac{{V}_{f}{K}_{mQ}R}{{V}_{r}{K}_{eq}}+\frac{{V}_{f}{K}_{mR}Q}{{V}_{r}{K}_{eq}}+ABC+\frac{{V}_{f}PQR}{{V}_{r}{K}_{eq}}+\alpha \left|{V}_{f}-{V}_{f0}\right|}$$

with the adjustment term $\alpha \left|{V}_{f}-{V}_{f0}\right|$ in the denominator, $\alpha$ is a defined number, ${V}_{f0}$ is the PPDK maximum rate in the forward direction used in the in vitro reconstitution and ${V}_{f}$ is the PPDK maximum rate in the forward direction in the model.

This particular model was built because, although the previous model could predict fairly well the final flux when PGAM and ENO activities were varied, it overestimated the flux when PPDK activity was varied. However, the previous model predicted the flux well, with the enzyme parameters used in the in vitro reconstitution. Therefore, an adjustment term should be added, in order to decrease PPDK rate with $\alpha$. Also, as ${V}_{f}$ of PPDK is equal to ${V}_{f0}$ when PGAM’s or ENO’s activity is varied, $\alpha$ is multiplied by ${V}_{f}-{V}_{f0}$, so that the adjustment term to be zero when ${V}_{f}={V}_{f0}$ and the flux predictions are not modified in these two cases mentioned above. Also, to ensure that the adjustment term is positive, we used the absolute value $\left|{V}_{f}-{V}_{f0}\right|$.

To determine the value of $\alpha$, first a range of values from 0 to 4*10^6^ with steps of 10^6^ is assessed. Then the range and the steps are reduced, from 10^6^ to 1, until we obtain better results for RMSE, and coefficient of determination (R^2^) between the predicted and experimental data. The equation for R^2^ is given below:

7 $${R}^{2}= 1- \frac{\sum_{i=1}^{n}{({Y}_{i}-{\widehat{Y_{i}}})}^{2}}{\sum_{i=1}^{n}{({Y}_{i}-\overline{Y})}^{2}}$$

with ${Y}_{i}$ and ${\widehat{Y_{i}}}$ respectively the observed and predicted values, n being the total number of values and i = 1, 2…*n*.

It is important to note that this parameter $\alpha$ has no biological significance and is determined by a data-driven learning method, hence the name “grey box” for this model.

### Model comparison

To compare accuracy of the models, RMSE, R^2^ and AIC are assessed for the experimental dataset (Table S2). The same statistical metrics are used to evaluate their generalization ability with the test dataset (Table S3).

AIC measures the quality of the model by taking into account its complexity. Additionally, as the ratio “number of data-number of parameters” is less than 40, a corrected AIC is calculated as follows^20,34^:

8 $$AIC = 2*k+n*ln\left(\frac{SSE}{n}\right)+\frac{2*k*\left(k+1\right)}{n-k-1}$$

with k being the number of parameters, SSE the Sum of Square Errors and n the number of data.

Furthermore, to assess the generalization ability of the models, a comparison of RMSE, R^2^ and MAE is made on the previous test set (Table S3).

### Flux control analysis

For purposes of analyzing the pathway flux control and identifying the key enzymes involved in the flux control, the flux control coefficient of each enzyme (${C}_{E}^{J}$) is calculated with each model (ANNs and metabolic networks). This measure, generally used in Metabolic Control Analysis (MCA), allows us to assess quantitatively the impact of the enzyme on the pathway flux^15–17^. Here, ${C}_{E}^{J}$ is determined in an analytical way using the formula mentioned below (9):

9 $${C}_{E}^{J}= \frac{\partial J}{\partial x}*\frac{{x}_{0}}{{J}_{0}}$$

where $J$ is the flux and x is either the enzyme activity in the case of ANNs or the rate of the reaction catalyzed by the enzyme in the case of metabolic networks (COPASI), multiplied by a scalar factor $\frac{{x}_{0}}{{J}_{0}}$ which represents the reference values of enzyme activity/reaction rate and pathway flux.

## Application and results

### ANN modeling of the second part of glycolysis

**Figure 4:**
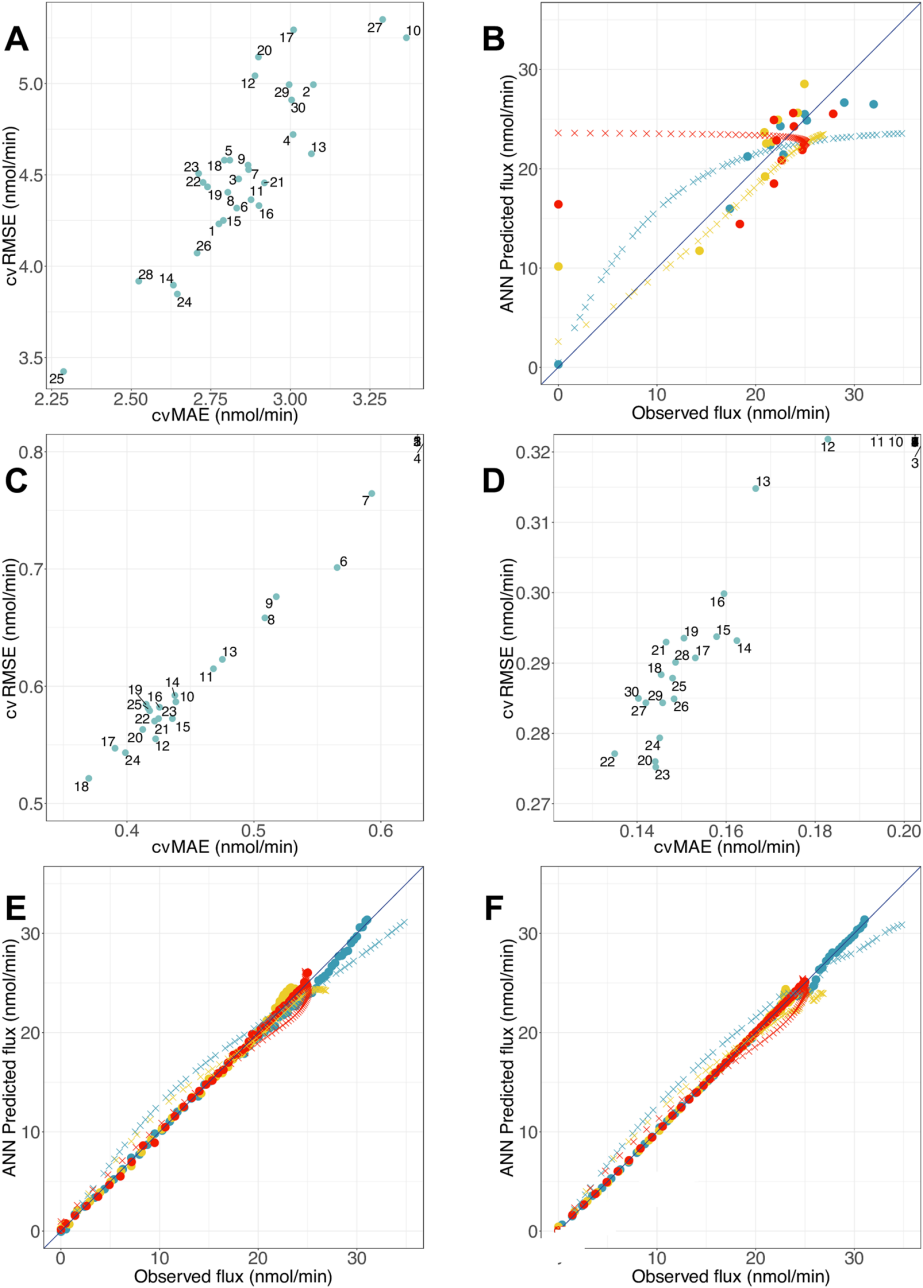
ANN model selections and flux predictions. (**A**) cvRMSE and cvMAE for the first dataset and using NeuralNet package and log activation function. The numbers represent the number of HUs. (**B**) Flux prediction with the best ANN model with 1 HU. Training: cvRMSE = 4.23 nmol·min^−1^, cvMAE = 2.78 nmol·min^−1^, cvR^2^ = 0.71 and Test: RMSE = 1.56 nmol·min^−1^, MAE = 1.24 nmol·min^−1^, R^2^ = 0.97. (**C**, **D**) cvRMSE and cvMAE for the second dataset and using NeuralNet package and tanh activation function (**C**) or Nnet package and log activation function (**D**). The numbers represent the number of HUs. (**E**) Flux prediction with the best ANN model using NeuralNet, tanh activation function and 18 HUs for the training set (circles) and test set (crosses). Training: cvRMSE = 0.52 nmol·min^−1^, cvMAE = 0.37 nmol·min^−1^, cvR^2^ = 1 and Test: RMSE = 1.61 nmol·min^−1^, MAE = 1.37 nmol·min^−1^, R^2^ = 0.98. (**F**) Flux prediction with the best ANN model using Nnet, log activation function and 23 HUs for the training set (circles) and test set (crosses). Training: cvRMSE = 0.28 nmol·min^−1^, cvMAE = 0.13 nmol·min^−1^, cvR^2^ = 1 and Test: RMSE = 1.69 nmol·min^−1^, MAE = 1.47 nmol·min^−1^, R^2^ = 0.98. Colored circles/crosses refer to the various levels of enzyme activity: PGAM (blue), ENO (yellow) or PPDK (red) for the training/test set.

First, we model the second part of *E. histolytica* glycolysis using the black-box modeling approach with ANN models and the first experimental dataset (Table S1, Fig. 4A,B) or the second experimental dataset (Table S2, Fig. 4C–F). For the first dataset, the evaluation of RMSE in cross-validation (cvRMSE) and MAE in cross-validation (cvMAE) shows a fluctuation of the error values when the number of HUs is varied and allows the identification of the best ANN model, presenting the lowest cvRMSE and cvMAE values. Also, the calculation of N_h_ gives a maximum value of 4 ($\alpha$ = 2), making it possible to identify the best model, regarding cvRMSE and cvMAE, with a number of HU equal to 1 (Fig. 4A). By comparing the ANN predicted fluxes with the experimental ones, we observe that this model can predict rather well the flux of the pathway for the training set, especially at high values of flux (Fig. 4B), and even if the calculated errors remain high (cvRMSE = 4.23 nmol·min^−1^, cvMAE = 2.78 nmol·min^−1^). The prediction of the test set shows that the model predicts the flux better when PGAM or ENO activity is varied, than when PPDK’s activity is varied. This can be explained by the small experimental data number in the training set, which is derived from experimentally controlled conditions. We built other ANN models with the NeuralNet package and tanh activation function and Nnet package, but the predictions are less good than those of previous models, with lower R^2^ in cross-validation (cvR^2^) and respectively, cvRMSE = 4.47 nmol·min^−1^ and cvMAE = 2.84 nmol·min^−1^, for the first one and cvRMSE = 4.56 nmol·min^−1^ and cvMAE = 2.66 nmol·min^−1^ for the second one (Fig. S1).

Afterwards, we built another ANN model, this time using the second dataset, corresponding to the data from the fitting curves obtained from the experimental points in the first dataset. From the two packages used, we notice that, with NeuralNet and tanh activation function, it is easier to identify the optimal number of HUs, which is 18, but this is not the case with the Nnet package, where the models with 22 and 23 HUs present a better cvMAE or a better cvRMSE (Fig. 4C,D). As RMSE is the most used model selection criterion of both, we use 23 HUs for the second model with the Nnet package. The comparison of these two models shows their ability to simulate the metabolic pathway, with better results for the Nnet model (Fig. 4E,F). Also, the calculation of N_h_ gives a maximum value of 23 ($\alpha$ = 2); thus, both models comply with the limit set by the equation.

However, in order to select the best model and ensure that it is not too specific to our second dataset, we used the test set from the most performing COPASI model (Table S3), and predicted the final flux with our two ANN models. The NeuralNet model produced better results, with RMSE = 1.61 nmol·min^−1^ and MAE = 1.37 nmol·min^−1^, compared to the Nnet model. These results suggest that this novel black-box approach, using ANN, is relevant for constructing metabolic pathways from experimental data, with better predictions when working with bigger datasets, whether it be with NeuralNet or Nnet package.

### Design of metabolic network with the white-box approach

After the modeling phase using the black-box method approach, we focused on the white-box approach and designed mechanistic models with COPASI. The first COPASI model we used was that of Moreno-Sanchez^8^; although it was created in GEPASI, we were able to work with this model on COPASI (Fig. S2A-C). The steady-state flux predicted with this model converged around 16.6 nmol·min^−1^ for the three enzymes, with a flux that decreased for PGAM and increased for ENO and PPDK during simulation time (Fig. S2A). This result was lower than the experimentally measured result (27 nmol·min^−1^)^8^. As for the prediction of metabolite concentrations, after one hour simulation time, 2PG was at 139.78 µM, PEP at 6.08 µM and Pyr at 8.31*10^–3^ µM (Fig. S2B). Here also the predicted concentrations were higher than the experimentally measured results, with a concentration of 2PG at 58 ± 29 µM and PEP at 37 ± 16 µM (Pyr experimental concentration was not available) in the previous work^8^. Furthermore, analysis of the predicted flux when enzyme activities were varied showed quite good prediction of the flux for PGAM and PEP, but not for PPDK, which showed RMSE of 4.33 nmol·min^−1^ (Fig. S2C).

**Figure 5:**
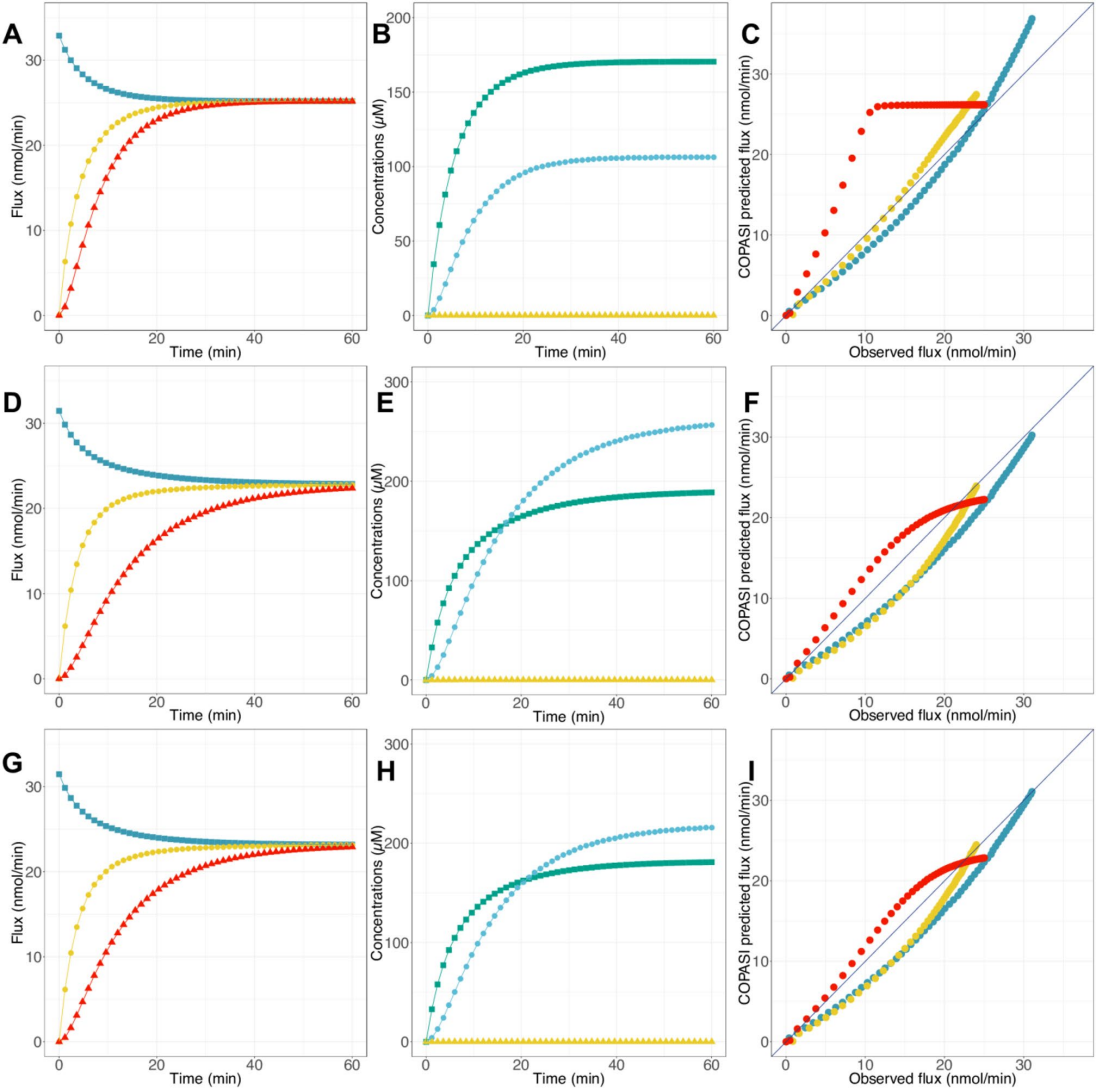
Flux and metabolite concentration predictions with COPASI models. (**A**, **D**, **G**) PGAM (blue squares), ENO (yellow circles) and PPDK (red triangles) flux predicted as function of time with the adjusted Moreno-Sanchez model (**A**), the model containing UUBB equation (**D**) and the improved model containing UUBB equation (**G**). (**B**, **E**, **H**) 2PG (green), PEP (blue) and Pyr (yellow) concentration predicted with the adjusted Moreno-Sanchez model (**B**), the model containing UUBB equation (**E**) and the improved model containing UUBB equation (**H**). (**C**, **F**, **I**) Flux predictions by the adjusted Moreno-Sanchez model (**C**), the model containing UUBB equation (**F**) and the improved model containing UUBB equation (**I**). Circle colors refer to the various levels of enzyme activity: PGAM (blue), ENO (yellow) or PPDK (red).

The results of this first model clearly indicate that the studied metabolic pathway can be modeled with COPASI as a biochemical network using different kinetic parameters and equations, but it needs to be fine-tuned to be more accurate in terms of predictions. The primary modification made in this model concerned the *V*_*max*_ values and the metabolite concentrations. Indeed, we replaced these values with those used in the experimental conditions at a pseudo steady-state (see Tables 1–3 and Fig. 5A–C). These changes have the effect of increasing the predicted fluxes and metabolite concentrations, in particular with a flux of 25.2 nmol·min^−1^ closer to the experimental value (Fig. 5A). As for the metabolite concentrations, they were still higher than those measured experimentally (Fig. 5B). The comparison between the predicted and observed fluxes revealed an enhancement of the predictive capability of our model with RMSE = 3.39 nmol·min^−1^ and R^2^ = 0.88 (Fig. 5C), emphasizing the importance of using appropriate parameters in the model.

However, this second model presents a poorer ability to predict the flux when PPDK activity is varied. For this reason, we decided to improve it by modifying the PPDK kinetic equation only and replace the Bi Bi Ping Pong kinetic equation used in the preceding models with the more precise Uni Uni Bi Bi kinetic equation defined by Varela-Gómez et al.^14^ (Fig. 5D–F). As some kinetic parameters (*K*_*i*_ and *K*_*ii*_) were not characterized experimentally, they were arbitrarily fixed at 1,000 µM (see Table 4). This last model yielded a slight decline of reaction fluxes to around 22 nmol·min^−1^ and higher metabolite concentrations than experimentally determined (Fig. 5D,E). Interestingly, we noted an improvement of flux predictions when enzyme activities were varied (RMSE = 2.43 nmol·min^−1^ and R^2^ = 0.94), in particular in the case of PPDK activity variation (Fig. 5F). Therefore, this second attempt to refine the COPASI model revealed that beyond the use of appropriate parameters, our model has to include precise kinetic equations to be more efficient.

As we said before, some parameters are not yet defined experimentally; therefore, we use COPASI Parameter Estimation task to estimate these kinetic parameters. The best results are obtained with the Particle Swarm optimization method, with a cost function of 771.135; the optimized values of *K*_*i*_ and *K*_*ii*_ are presented in Table S4. It is worth noting that the cost function value remains high, suggesting a failure of COPASI to estimate parameters better. This could be due to the high number of values to be parameterized and the low number of experimental data. Besides, these parameterized values have no physiological meaning, since they are in the molar range, and could be explained by the negligible impact of the parameterization with COPASI. Simulations run for one hour and fluxes and concentrations are analyzed again (Fig. 5G–I). We notice no significant change between the initial model and the optimized one. For the most part, the fluxes are increased: PGAM flux is at 23.4 nmol·min^−1^ and ENO flux at 22.9 nmol·min^−1^, except for PPDK flux which is at 21.3 nmol·min^−1^, while metabolite concentrations are greater than their experimental values (Fig. 5G,H). In general, we notice a minor enhancement of flux predictions with this optimized model (Fig. 5I). These findings suggest that the white-box modeling approach, through COPASI modeling, stands as a conventional method of choice to build consistent in silico models of metabolic pathways and this, despite the fact that, in our case, metabolite concentrations are poorly predicted even after the parameterization of the kinetic constants.

Besides, other approximative models, with lin-log approximation kinetics and Liebermeister kinetics, could have been evaluated^35,36^. Consequently, we built a model including the approximative lin-log equation (see modeling details in the legend of Fig. S3). Despite simplifying the rate equation by using lin-log kinetics, the model gives results comparable to the previous white-box model, with RMSE = 4.8 nmol·min^−1^ and R^2^ = 0.78 (Fig. S3C). Another model using the simpler modular rate law from Liebermeister^36^ is built (see modeling details in the legend of Fig. S4). This model has the immediate effect of simplifying the rate equation for PPDK and allows good prediction of flux (26 nmol·min^−1^) in the experimental conditions (Fig. S4A). However, results show that metabolite concentrations are still overestimated and the model presents a lower predictive capacity compared to the previous models, with RMSE = 4.03 nmol·min^−1^ and R^2^ = 0.87 (Fig. S4B,C). Both models, with lin-log approximation kinetics or Liebermeister kinetics, display the same dynamics, with better flux predictions when PGAM’s or ENO’s activity is varied than when PPDK’s activity is varied. Together, these results reveal that there are some aspects of PPDK kinetics that are not completely modeled by these different mechanistic approaches.

### The grey-box modeling approach

**Figure 6:**
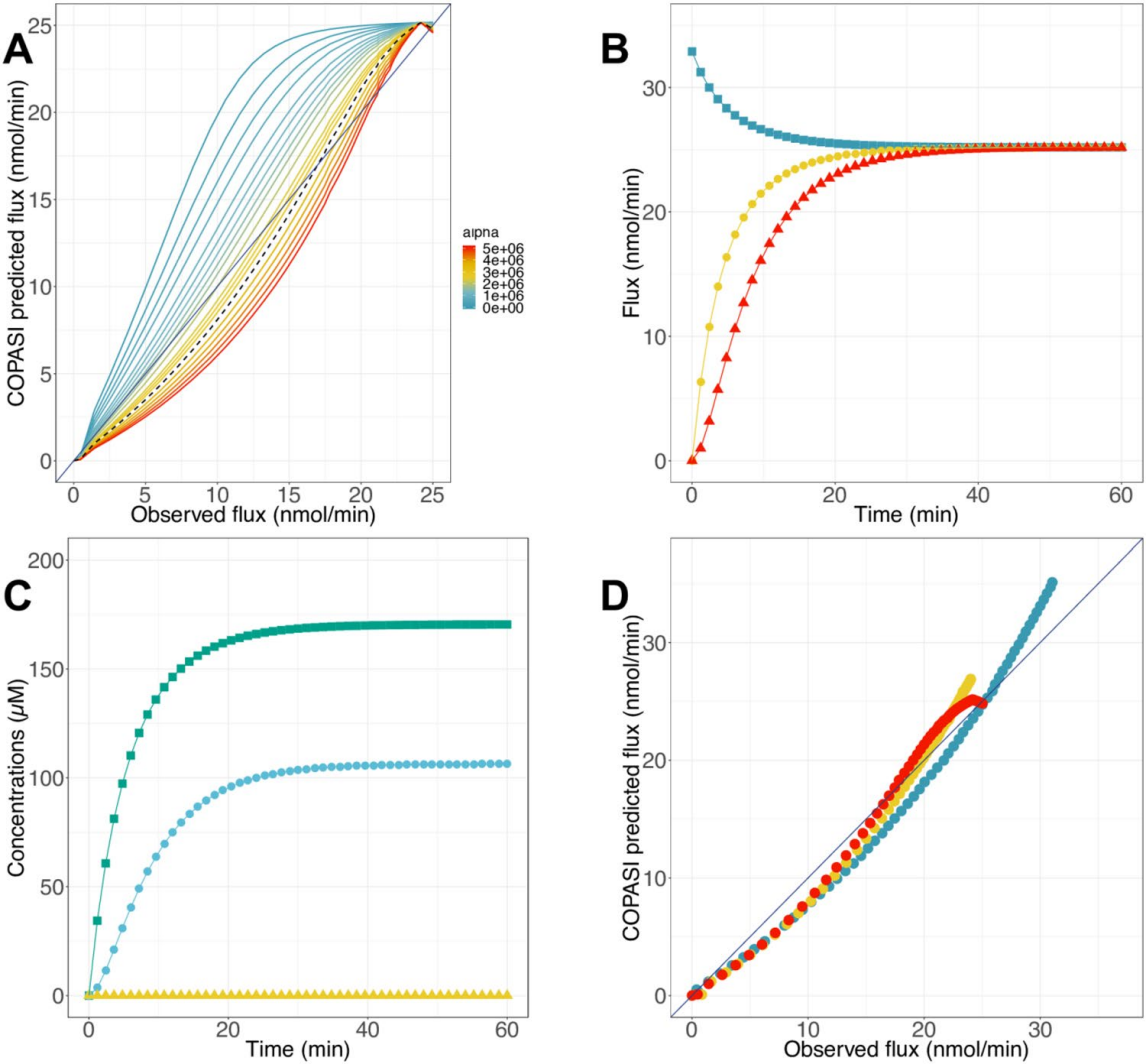
Flux and metabolite concentration predictions with COPASI model with an added adjustment term. (**A**) Flux predictions by the model when PPDK activity is varied. Dotted line: curve obtained with the best adjustment term (3,088,970). **(B**) PGAM (blue squares), ENO (yellow circles) and PPDK (red triangles) fluxes predicted as function of time. (**C**) 2PG (green), PEP (blue) and Pyr (yellow) concentration predictions. (**D**) Flux predicted by the model. RMSE = 1.71 nmol·min^−1^, MAE = 1.47 nmol·min^−1^, R^2^ = 0.98. Circle colors refer to the varied enzyme activity: PGAM (blue), ENO (yellow) or PPDK (red).

Based on our previous experiences, the major hurdle in the second part of glycolysis modeling is the third reaction catalyzed by PPDK. Then, we investigate the use of a novel approach called the grey-box modeling approach, consisting of using an adjustment term ($\alpha \left|{V}_{f}-{V}_{f0}\right|$) in the kinetic equation of PPDK. In order to define the optimal value of $\alpha$ in the adjustment term, we test a range of values from 0 to 5*10^6^ and identify the best value $\alpha$ around 3.09*10^6^; below this value, the flux is overestimated, and above, the final flux is underestimated (Fig. 6A). Also, no changes are made to the predicted flux when PGAM or ENO activity is varied (Fig. S5).

Again, simulations were performed over one hour with COPASI and the results of prediction are shown (Fig. 6B–D). We observed that the fluxes were around 25 nmol·min^−1^ as in a previous model (Figs. 5A and 6B), and consequently closer to the experimental value. In regards to the metabolite concentration predictions, they were also similar to those predicted with the previous model and were still higher than expected (Fig. 5B and 6C). Remarkably, a significant improvement of flux predictions was achieved, notably when PPDK activity was varied, compared to all other models analyzed before (Figs. 5C, F, I and 6D). Collectively, these results validate the use of the adjustment term in the kinetic equation to improve the metabolic pathway model built with COPASI.

### Model comparison and reliability

Following the design of the second part of glycolysis using three modeling approaches, we assess the reliability of each approach and proceed to their comparison. Also, for an easier understanding of the following results, the properties of each model are summarized in Table 5.

**Table 5:** List of the main properties of each model.

Model^a^	Name	Specificity^b^	Number of parameters	Based on…
0	Moreno-Sanchez model	See^8^	20	Experimental kinetic data
1	Adjusted Moreno-Sanchez model	Respects the experimental conditions at a pseudo steady-state	20	
2	ANN model (NeuralNet, log, HU = 1)	Only uses the experimental dots	6	Enzyme activities and final flux data
3	ANN model (NeuralNet, tanh, HUs = 18)	Uses data from the fitting curves	91	
4	UUBB model	Use of UUBB equation for PPDK^c^	29	Experimental and fitted kinetic data
5	UUBB model optimized	Uses the UUBB equation for PPDK with optimized parameters^c^	29	
6	Model with an added adjustment term	PPDK equation with an added adjustment term^c^	21	Experimental kinetic data + adjustment term

^a^Only the best models from each approach are kept.

^b^For a complete description of the modeling process, see the “Methodology” section.

^c^Respect of pseudo steady-state experimental conditions.

**Figure 7:**
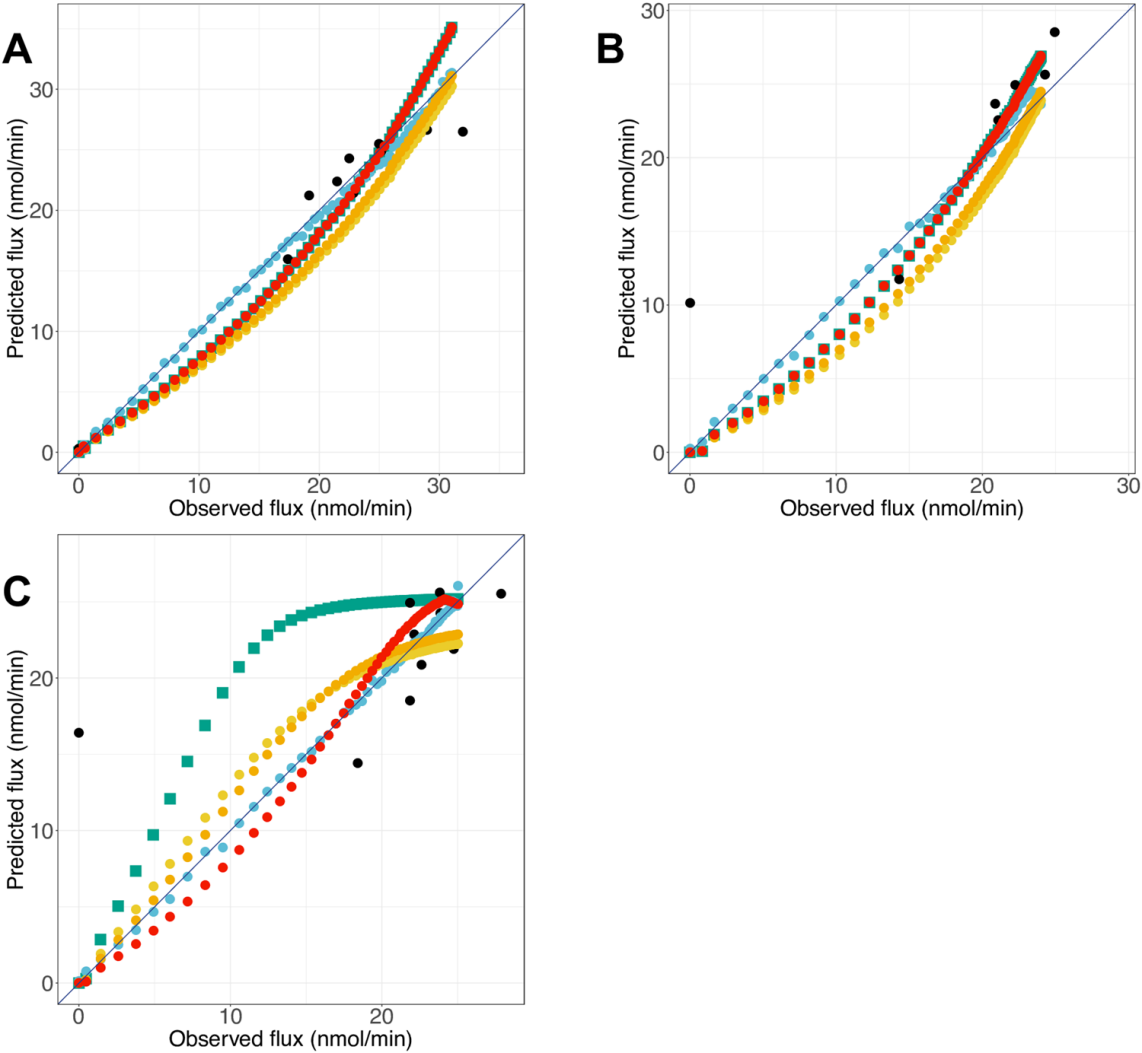
Comparison of flux predictions and experimental flux for all models. Flux predictions by the model, when PGAM activity (**A**), ENO activity (**B**) or PPDK activity (**C**) is varied. Colors refer to the model used: Model 1 (green squares), Model 2 (black circles), Model 3 (blue circles), Model 4 (yellow circles), Model 5 (orange circles) and Model 6 (red circles).

By comparing the predicted fluxes to their experimental values, we found that all models, from Models 1–6, worked well for predicting the final flux when activity of PGAM varies (Fig. 7A). When ENO activity is varied, we notice that Model 2 does not perform well, particularly for the low values, for which the model overestimates the final flux (Fig. 7B). Besides, for these two enzymes we note that Models 1, 4 and 5 from the white-box approach and Model 6 from the grey-box approach underestimate the flux when activity of PGAM or ENO is varied, with a gap that seems smaller in the case of the grey-box approach. As expected, dots from Model 3 are practically aligned with the first bisector, suggesting an almost perfect flux prediction with this model (Fig. 7A,B). Lastly, the variation of PPDK activity shows the greatest effect on model prediction. We observe that Model 2, as well as Model 1, are the two models that have the most difficulty in predicting flux under these conditions (Fig. 7C). Indeed, they overestimate the flux when PPDK activity is varied; this was also the case for Models 4 and 5, but with a smaller difference between the predicted and observed values. In contrast, fluxes are closely predicted with Models 3, 5 and 6. These results indicate that these models are suitable to simulate our studied metabolic pathway and that we can count on their reliability for the analysis of the flux in the second part of glycolysis, at least for an overall flux ranging from 0 to 30 nmol·min^−1^.

The analysis of the statistics for each model reinforced the results obtained before (Table 6). Indeed, all models exhibited a fairly low RMSE under 3 nmol·min^−1^ and a high R^2^, around 0.98, when PGAM activity was varied. When ENO activity was varied, almost all models predicted the flux with a good RMSE under 3 nmol·min^−1^ and R^2^ above 0.97, except for Model 2. However, when PPDK activity was varied, Models 0, 1 and 2 showed the weakest results, with RMSE above 5 nmol·min^−1^ and a R^2^ under 0.9. Only the three models mentioned above (Models 3, 5 and 6) yielded good results with a low RMSE and a high R^2^ value. These results corroborated those obtained earlier. Interestingly, the calculation of AIC allows the establishment of a ranking of models (from the best to less good): Model 2 > 3 > 6 > 5 > 4 > 1 > 0 (Table 6). Model 2, which has the lowest AIC, proved to be a poor model for flux prediction. Conversely, Model 3, that gives the best results in terms of RMSE, MAE and R^2^ presents a good AIC. We also notice that the second-best model in flux prediction (Model 6) also presents a low AIC value.

**Table 6:** Comparative table of statistical metrics of each model for the training set (Table S2).

	R^2^	RMSE	MAE	AIC	PGAM		ENO		PPDK	
					R^2^	RMSE	R^2^	RMSE	R^2^	RMSE
Model 0	0.85	4.33	3.17	584.74	0.98	2.42	1	2.41	0.71	6.75
Model 1	0.88	3.39	2.48	494.39	0.98	2.02	0.98	1.78	0.8	5.27
Model 2^a^	0.71	4.23	2.78	99.5	0.94	2.19	0.78	4.02	0.41	5.71
Model 3^a^	1	0.52	0.37	124.21	1	0.62	1	0.62	1	0.22
Model 4	0.94	2.43	2.1	396.72	0.98	2.96	0.97	2.3	0.94	1.94
Model 5	0.95	2.06	1.7	336.05	0.98	2.59	0.98	1.89	0.96	1.6
Model 6	0.98	1.71	1.47	244.71	0.98	2.02	0.98	1.78	0.99	1.22

RMSE and MAE are in nmol·min^−1^.

^a^For these models, (cv)RMSE and (cv)R^2^ are calculated.

Subsequently, in order to evaluate the generalization ability of our models, we predict the flux with the test set (Table 7). Many models do not have an adequate ability of generalization; nevertheless, Model 6 from the grey-box approach stands out from the others. Indeed, it is the only model able to predict the flux very well from new data, regardless of the enzymatic activity that is varied. Model 0 and 1 can predict the flux well, except when PPDK activity is varied. Also, AIC calculations identify Model 6 as the best one to generalize (AIC = − 486.7), since Model 3 presents higher RMSE, MAE and AIC value (AIC = 539.06). These results confirm the reliability of the three approaches for the analysis of the flux in the second part of glycolysis, with a preference for Model 6, which offers the best compromise between precision and complexity.

**Table 7:** Comparative table of statistical metrics of each model for the test set (Table S3).

	R^2^	RMSE	MAE	AIC	PGAM		ENO		PPDK	
					R^2^	RMSE	R^2^	RMSE	R^2^	RMSE
Model 0	0.86	3.71	2.15	527.32	1	0.88	0.99	1.42	0.59	6.26
Model 1	0.89	2.78	1.06	421.76	1	0.02	1	0.11	0.72	4.87
Model 2	0.52	5.54	3.89	642.46	0.99	4.04	1	5.71	0.99	4.17
Model 3	0.98	1.61	1.37	539.06	0.98	2.12	0.99	1.32	0.98	1.26
Model 4	0.96	2.73	2.51	439.52	1	2.68	1	2.89	0.93	2.63
Model 5	0.97	2.19	2	357.26	1	2.17	1	2.3	0.95	2.08
Model 6	1	0.23	0.13	-486.7	1	0.02	1	0.11	1	0.39

### Identification of the main controlling enzymes of the pathway

After establishing three types of models for the considered metabolic pathway, we determined the enzyme ${C}_{E}^{J}$ with each model. These coefficients are calculated at a pseudo steady-state flux to Lac (Table 8) or at physiological metabolite concentrations (Table S5) at the reference or basal level of enzyme activity of 75 mU PGAM, 328.5 mU ENO and 196.5 mU PPDK. Each ${C}_{E}^{J}$ provides a quantitative measurement of the enzyme effect on the pathway flux. The closer the coefficient is to 1, the higher the enzyme impact on the flux. Thus, this coefficient differs from the concept of rate-limiting enzyme, which is commonly defined as the enzyme which catalyzes the slowest step in the pathway and corresponds to a qualitative evaluation of the enzyme impact on the pathway flux^15–17^.

**Table 8:** Flux control coefficient determination.

Model	PGAM	ENO	PPDK
Experimentally determined^8^	0.72	0.11	0.13
Model 0	0.79	0.21	0.0025
Model 1	0.75	0.21	0.04
**Model 2**^a^	**0.4**	**0.33**	**0.22**
**Model 3**^a^	**0.61**	**0.12**	**0.25**
Model 4	0.70	0.2	0.1
Model 5	0.71	0.2	0.09
Model 6	0.75	0.21	0.002

^a^For these models, ${C}_{E}^{J}$ are determined manually.

As we can see, at a pseudo steady-state flux to Lac, the enzyme that exerted the greatest control on the final flux is PGAM (0.65 ± 0.2), then ENO (0.18 ± 0.04) and PPDK (0.07 ± 0.1) which showed the least control on the flux (Table 8). The predicted values by the different models are within the same interval as those experimentally determined by pathway reconstitution^8^. Similar results were obtained with all models at physiological metabolite concentrations (Table S5). From these findings, we can conclude that the main controlling enzymes of the second part of glycolysis in *E. histolytica* are PGAM and, to a lesser extent, ENO and PPDK exert low or no control over the pathway flux.

**Figure 8:**
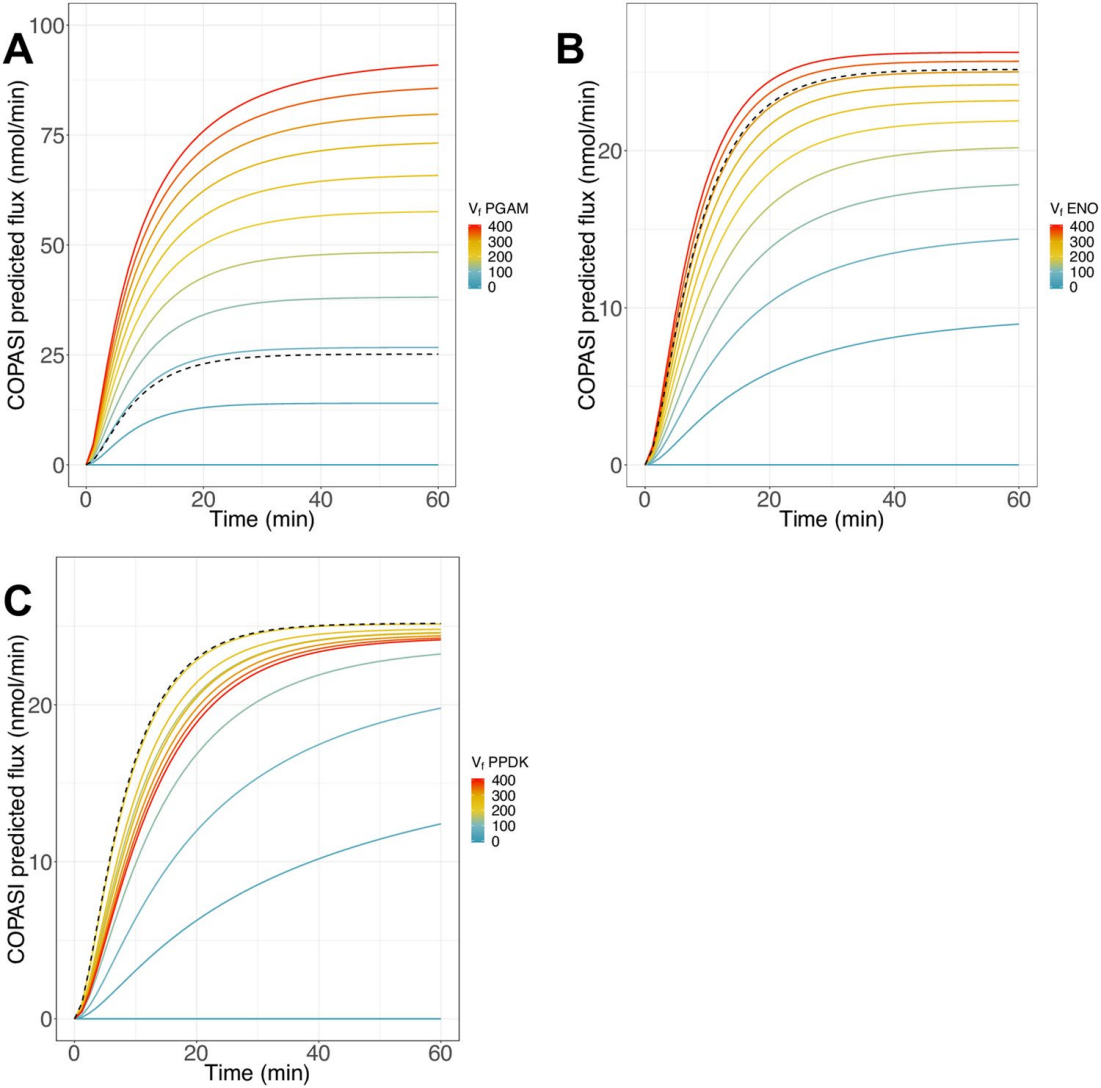
Effect of enzyme variation on the pathway flux. Pathway flux predicted with the model with the added adjustment term, when PGAM activity (**A**), ENO activity (**B**) or PPDK activity (**C**) is varied. Dotted curves: fluxes obtained at the quasi steady-state to Lac. *V*_*f*_ of PGAM, ENO and PPDK are in mU.

In addition, we varied the enzyme activity from 0 to 400 mU and observed the final flux during the first hour of simulation using the COPASI model with the adjustment term (Fig. 8). When PGAM was varied, the flux went from 0 to 90.93 nmol·min^−1^ (Fig. 8A) and when ENO was varied, the flux went to 26.26 nmol·min^−1^. By contrast, PPDK activity variation did not affect the final pathway flux very much, which went to 24.13 nmol·min^−1^ at 400 mU of PPDK. These results were consistent with previous ${C}_{E}^{J}$ calculations showing that PGAM and ENO are indeed the two main controlling enzymes of the pathway.

## Discussion

### Relevance of the white- grey- and black-box approach for the modeling of metabolic pathways

In this work, we model the second part of the glycolysis pathway of *E. histolytica* using three approaches: the white-, grey- and black-box approach, and we highlight their ability to predict the final flux. Many comparative studies are made in other fields to evaluate the relevance of using either of the three methods, and point out that the method depends on the problems encountered^37–39^. In the case of energy model building, Li and Wen showed that simplified grey-box models are better as practical building models, compared to white-box models that require numerous parameters^38^. In another study, the black-box models outperformed the other two models for the modeling of thermal simulation in a particular environment^39^.

Here, the first approach is based on the use of kinetic parameters and equations and is related to the widely used method known as kinetics-based (or dynamic) modeling for industrial applications such as the production of molecules of interest, development of de novo synthesis pathways or understanding of microorganism metabolism^40–42^. This method can provide accurate predictions; however, it requires numerous parameters and good knowledge of mechanistic rate laws; hence the need to develop new strategies of modeling when we do not have access to this information^43,44^.

Despite the use of a more complex kinetic equation in the kinetic models, the results were not satisfactory; consequently, we used a simplified kinetic equation with an adjustment term in the grey-box approach. This is the first time this method is applied to enhance performance of a metabolic pathway kinetic model. In other studies found in the literature, the unknown kinetic constants are parameterized or the kinetic equations can be approximated^36,45–47^. The present approach has some major advantages as it needs less parameters than the white-box approach, and it uses simplified kinetic equations that are biochemically plausible.

Finally, we used a novel black-box approach and built an ANN model with experimental data. As previously mentioned, ANN is generally used in biology to solve classification problems, for example, to classify lung carcinomas^48^, but it has rarely been used to model a metabolic pathway^49–52^. A recent study applied a similar technique to model the first part of glycolysis, and showed the success of this technique for predicting the flux^53^. This last approach is characterized by its rapidity; however, it requires a large number of experimental data to be sufficiently effective.

Together, the approaches we describe here may be beneficial for modeling other metabolic pathways, depending on background information including “raw” experimental data, kinetic parameters and kinetic equations.

### Factors impacting model performance

During this study, we relied on three main statistical metrics (RMSE, MAE and AIC) to evaluate model performance. The results revealed that different criteria are important and impact the value of these metrics and thus the model performance itself. Among these criteria, we identified the size of the dataset, but also the choice of the activation function (log or tanh) and the number of HU in our ANN models. Indeed, having a large number of high-quality datasets is essential to obtain a good ANN, and one challenge here would be to avoid over-fitting^54,55^. Other studies bring out the importance of the size of the input sequence during the analysis of the DNA sequence, to increase model performance^56^. Also, they reveal the relevance of neural network architecture, proposing the design of multi-task neural networks with multiple output variables^57^. These factors raise new questions about the use of ANN to model metabolic pathways, and can be subjected to further investigation concerning the number of inputs and outputs to include in our model, to make it more efficient.

As we said earlier, to predict accurate results COPASI models need extensive data, such as kinetic parameters and equations. Our results reveal the impact of the kinetic equation on the final flux prediction. The impact of the kinetic equation on the model predictions depends on the complexity of the model and on the flux control coefficient of the enzyme. When the enzyme has high ${C}_{E}^{J}$, variations of its rate equation or small variations in the kinetic constants or *V*_*max*_ greatly impact the predicted pathway flux (e.g. PGAM in Fig. 8). In contrast, rate equation variations of a low controlling enzyme (such as PPDK) have less impact on the flux. It would be interesting to test in the models the influence of the lack of regulatory feedback on the enzyme that has the highest control, as was done in the Moreno-Sanchez et al. study^8^ focusing on PGAM. As was described in that paper, the lack of those regulatory effects renders the predictive power of the model ineffective. Therefore, regulatory properties on high controlling enzymes can drastically modify the model predictions. Furthermore, the question of which kinetic equation to use in the pathway remains a real topic in research today. Kim et al. review all kinetic rate expressions used in the kinetic model, from mechanistic expressions (Michaelis–Menten and Hill rate laws equations) to approximate kinetic equations (lin-log kinetics, modular rate laws…)^58^. These approximate kinetic equations have the advantage of simplifying the modeling, but they cannot help with estimating the parameters. Moreover, particular attention is given to the kinetic parameters that need to be as close as possible to in vivo kinetics. This can be done during enzyme analysis by bringing the in vitro conditions closer to the in vivo conditions^58^. Therefore, the consideration of these different factors may impact the process of model design but also the upstream research that is done to study metabolic pathways in a particular organism.

### Possible model optimizations

Although we have almost accurate prediction results, we can consider additional improvements of the different models. Actually, as this analysis is only made on the second part of glycolysis, it could be envisioned to merge it with the first part of this metabolic pathway to investigate the changes in terms of ${C}_{E}^{J}$ and pathway flux control, and then compare the results to the previous ones, where the parts were modeled separately^8^. It would be interesting to have a detailed kinetic model of glycolysis in *E. histolytica* combined with other major metabolic pathways (glycogen metabolism, pentose phosphate pathway)^7^, to highlight the need to inhibit or not the main controlling enzymes identified here, as was done for cancer cells^59^. Also, the addition of genetic-level regulations could help to better understand parasite metabolism, as is done for *E. coli*^60^. However, in order to do this, we still need experimental data on gene expression and regulation in the parasite under conditions of infection.

Also, another way to optimize the models could be by parameter estimation of the unknown kinetic parameters in the UUBB equation. Here, we tried to estimate these parameters, defined first arbitrarily, but the parameter estimation results in very little improvement of the flux prediction with the use of the new estimated values. Actually, parameterization of kinetic constants can provide a mathematical solution to the problem with unrealistic values likely to be physiologically unlikely. Hence, the importance of performing parameter estimation with constraints, within intervals that may be possible in enzymes and may have physiological meaning (e.g.* K*_*m*_ or *K*_*i*_ values not surpassing the lower mM interval). This emphasizes again the need for more experimental data concerning the PPDK mechanism in in vivo conditions. Additionally, in kinetic models, parameters can be determined in two ways, as we have done, either one at a time or collectively; the only difference being that some parameters are often set to measured values^43,58^. We can also consider the use of different parameter estimation techniques. As demonstrated in a previous work, kinetic parameters can be estimated with the flux balance analysis constraint-based modeling approach, by integrating multi-omics data in the model (fluxomic, proteomic and metabolomics data)^61^. Consequently, additional work needs to be done involving this part of the modeling, to improve our white-box model using a UUBB equation; it would also be interesting to integrate the data from the grey-box approach into the next parameter estimation procedure.

### Biological insights

With the MCA method (${C}_{E}^{J}$) and with all models, we identified PGAM as the main controlling enzymes of the second part of glycolysis in this parasite, with a slight contribution of ENO. These results are supported by other studies conducted on this particular pathway^7,8^. Furthermore, it has been found by elasticity analysis, another experimental approach of MCA, that the group of enzymes from PPi-dependent phosphofructokinase to PPDK controls about 0.2–0.28 of the pathway flux of amoebal glycolysis^62^. Within this pathway section, PGAM is the enzyme with the lowest activity in the cell^7^, which may contribute to the better control observed. Additionally, novel enzyme inhibitors were recently identified and tested in vitro^63,64^. Therefore, these models may be an interesting subject of future research in which the inhibitor effect on the flux can be assessed.

## Conclusion

Be it for the purpose of designing new valuable enzymatic pathways for industrial-scale production of molecules of interest or designing new efficient drugs, metabolic pathway modeling remains a great challenge today^65–67^. Different techniques of modeling exist, including kinetic modeling, based on the use of kinetic parameters and equations that are not necessarily known or experimentally measured. Moreover, several machine learning-based methods are emerging for analysis of metabolic pathway modeling^68,69^.

In this study, our objective was to compare three different modeling approaches to model metabolic pathways and identify the main controlling enzymes of the pathway. To this end, we used an application example (lower part of glycolysis of a parasite) and obtained:

- The white-box approach, with the use of all known kinetic information about PGAM, ENO and PPDK. This method gave better results after the modification of the PPDK kinetic equation from ter-reactant reversible equation to UUBB equation (Training: R^2^ = 0.95, RMSE = 2.06 nmol·min^−1^ and MAE = 1.7 nmol·min^−1^ and AIC = 336.05 and Test: R^2^ = 0.97, RMSE = 2.19 nmol·min^−1^ and MAE = 2 nmol·min^−1^).
- The grey-box approach, with the kinetic equation with an added adjustment term for PPDK; this model was the best of our models (Training: R^2^ = 0.98, RMSE = 1.71 nmol·min^−1^, MAE = 1.47 nmol·min^−1^ and AIC = 244.71 and Test: R^2^ = 1, RMSE = 0.23 nmol·min^−1^ and MAE = 0.13 nmol·min^−1^).
- The black-box method, using the ANN method to predict the pathway flux. This model presents a low capacity of generalization since its low AIC (124.21) makes it one of the least preferred models here. Nonetheless, the speed and the low cost of this method make it interesting to develop. The model had a good predictive ability with Training: cvR^2^ = 1, cvRMSE = 0.52 nmol·min^−1^, cvMAE = 0.37 nmol·min^−1^ and AIC = 124.21 and Test: R^2^ = 0.98, RMSE = 1.61 nmol·min^−1^ and MAE = 1.37 nmol·min^−1^.

Also, all these models identified the same enzymes as the main controlling enzymes of the pathway: PGAM and ENO, PPDK not having much influence on the flux in *E. histolytica*.

Despite the need for further improvement, these models showed the relevance of the different methods for their future application in the field of metabolic pathway modeling and drug design, for in silico design starting from various backgrounds.

## Supplementary information

Supplementary information

## Data Availability

The datasets used in this study are fully included and described in the Additional file. The ANN and COPASI models built during the present study are available in the Github repository, https://github.com/ophelielt/Lo-Thong_et_al._White-box_grey-box_and_black-box_pathway_modeling.git. All data generated or analyzed during this study are included in this published article (and its Supplementary Information files).
