# Supplementary material for: Identification of flux checkpoints in a metabolic pathway through white-box, grey-box and black-box modeling approaches
Source: Sci Rep. 2020 Aug 10;10:13446. doi: 10.1038/s41598-020-70295-5 (PMC7417601; doi:10.1038/s41598-020-70295-5)
Supplement: Supplementary file 1 — Supplementary information [file 41598_2020_70295_MOESM1_ESM.docx]

**Supplementary Information**

# Identification of flux checkpoints in a metabolic pathway through white-box, grey-box and black-box modeling approaches

# Ophélie Lo-Thong^1,2^, Philippe Charton^1,2^, Xavier F. Cadet^3^, Brigitte Grondin-Perez^4^, Emma Saavedra^5^, Cédric Damour^4^, Frédéric Cadet^1,2^*

# ^1^ University of Paris, UMR_S1134, BIGR, Inserm, F-75015 Paris, France.

# ^2^ DSIMB, UMR_S1134, BIGR, Inserm, Laboratory of Excellence GR-Ex, Faculty of Sciences and Technology, University of La Reunion, F-97715 Saint-Denis, France.

# ^3^ PEACCEL, Artificial Intelligence Department, 6 square Albin Cachot, box 42, 75013 Paris, France.

# ^4^ LE2P, Laboratory of Energy, Electronics and Processes EA 4079, Faculty of Sciences and Technology, University of La Reunion, 97444 St Denis cedex, France.

# ^5^ Departamento de Bioquímica, Instituto Nacional de Cardiología Ignacio Chávez. Mexico City, 14080, Mexico.

# ^*^ Corresponding author

# E-mail: [frederic.cadet.run@gmail.com](mailto:frederic.cadet.run@gmail.com)

# Table S1. Measured pathway flux (*J_obs_*) for different sets of enzyme activities (experimental dots)

| **PGAM (mU)** | **ENO (mU)** | **PPDK (mU)** | ***J_obs_* (nmol·min^-1^)** |
| --- | --- | --- | --- |
| 0 | 328.5 | 196.5 | 0 |
| 36.02 | 328.5 | 196.5 | 17.37 |
| 51.05 | 328.5 | 196.5 | 19.17 |
| 58.05 | 328.5 | 196.5 | 22.82 |
| 62.93 | 328.5 | 196.5 | 21.48 |
| 70 | 328.5 | 196.5 | 22.5 |
| 75.11 | 328.5 | 196.5 | 25.17 |
| 83.08 | 328.5 | 196.5 | 24.96 |
| 90.08 | 328.5 | 196.5 | 28.97 |
| 108.2 | 328.5 | 196.5 | 31.95 |
| 75 | 0 | 196.5 | 0 |
| 75 | 71.78 | 196.5 | 14.3 |
| 75 | 143.27 | 196.5 | 21.07 |
| 75 | 200.74 | 196.5 | 20.95 |
| 75 | 250.9 | 196.5 | 21.69 |
| 75 | 286.49 | 196.5 | 20.88 |
| 75 | 328.42 | 196.5 | 22.25 |
| 75 | 372.63 | 196.5 | 24.26 |
| 75 | 458.36 | 196.5 | 24.93 |
| 75 | 328.5 | 0 | 0 |
| 75 | 328.5 | 77.54 | 18.39 |
| 75 | 328.5 | 115.9 | 21.85 |
| 75 | 328.5 | 134.95 | 22.63 |
| 75 | 328.5 | 155.18 | 22.11 |
| 75 | 328.5 | 174.25 | 24.72 |
| 75 | 328.5 | 186.15 | 23.87 |
| 75 | 328.5 | 197.09 | 21.85 |
| 75 | 328.5 | 213.29 | 23.8 |
| 75 | 328.5 | 232.13 | 27.85 |

The experimental dots from Fig. 2 of Ref. [8] were digitized to obtain the data shown in the table. For each dataset, only one enzyme was varied and the other two were kept constant.

# Table S2. Pathway flux (*J*) for different sets of enzyme activities (from fitting curves)

| **PGAM (mU)** | **ENO (mU)** | **PPDK (mU)** | ***J* (nmol·min^-1^)** |
| --- | --- | --- | --- |
| 0 | 328.5 | 196.5 | 0 |
| 0.93 | 328.5 | 196.5 | 0.50 |
| 1.36 | 328.5 | 196.5 | 0.38 |
| 3.21 | 328.5 | 196.5 | 1.40 |
| 5.11 | 328.5 | 196.5 | 2.42 |
| 7.01 | 328.5 | 196.5 | 3.43 |
| 8.91 | 328.5 | 196.5 | 4.42 |
| 10.82 | 328.5 | 196.5 | 5.35 |
| 12.72 | 328.5 | 196.5 | 6.26 |
| 14.62 | 328.5 | 196.5 | 7.12 |
| 16.52 | 328.5 | 196.5 | 7.95 |
| 18.42 | 328.5 | 196.5 | 8.75 |
| 20.32 | 328.5 | 196.5 | 9.51 |
| 22.22 | 328.5 | 196.5 | 10.26 |
| 24.12 | 328.5 | 196.5 | 11.02 |
| 26.02 | 328.5 | 196.5 | 11.80 |
| 27.92 | 328.5 | 196.5 | 12.49 |
| 29.82 | 328.5 | 196.5 | 13.20 |
| 31.72 | 328.5 | 196.5 | 13.91 |
| 33.62 | 328.5 | 196.5 | 14.55 |
| 35.52 | 328.5 | 196.5 | 15.15 |
| 37.43 | 328.5 | 196.5 | 15.74 |
| 39.33 | 328.5 | 196.5 | 16.37 |
| 41.23 | 328.5 | 196.5 | 16.90 |
| 43.13 | 328.5 | 196.5 | 17.45 |
| 45.03 | 328.5 | 196.5 | 18.02 |
| 46.93 | 328.5 | 196.5 | 18.57 |
| 48.83 | 328.5 | 196.5 | 19.10 |
| 50.73 | 328.5 | 196.5 | 19.59 |
| 52.63 | 328.5 | 196.5 | 20.03 |
| 54.53 | 328.5 | 196.5 | 20.61 |
| 56.43 | 328.5 | 196.5 | 21.12 |
| 58.33 | 328.5 | 196.5 | 21.68 |
| 60.23 | 328.5 | 196.5 | 22.07 |
| 62.13 | 328.5 | 196.5 | 22.53 |
| 64.04 | 328.5 | 196.5 | 22.97 |
| 65.94 | 328.5 | 196.5 | 23.34 |
| 67.84 | 328.5 | 196.5 | 23.78 |
| 69.74 | 328.5 | 196.5 | 24.21 |
| 71.64 | 328.5 | 196.5 | 24.64 |
| 73.54 | 328.5 | 196.5 | 25.00 |
| 75.44 | 328.5 | 196.5 | 25.44 |
| 77.34 | 328.5 | 196.5 | 25.88 |
| 79.24 | 328.5 | 196.5 | 26.10 |
| 81.14 | 328.5 | 196.5 | 26.44 |
| 83.04 | 328.5 | 196.5 | 26.75 |
| 84.94 | 328.5 | 196.5 | 27.14 |
| 86.84 | 328.5 | 196.5 | 27.46 |
| 88.75 | 328.5 | 196.5 | 27.78 |
| 90.65 | 328.5 | 196.5 | 28.15 |
| 92.55 | 328.5 | 196.5 | 28.47 |
| 94.45 | 328.5 | 196.5 | 28.75 |
| 96.35 | 328.5 | 196.5 | 29.08 |
| 98.25 | 328.5 | 196.5 | 29.40 |
| 100.15 | 328.5 | 196.5 | 29.69 |
| 102.05 | 328.5 | 196.5 | 29.99 |
| 103.95 | 328.5 | 196.5 | 30.29 |
| 105.85 | 328.5 | 196.5 | 30.57 |
| 107.75 | 328.5 | 196.5 | 30.88 |
| 109.22 | 328.5 | 196.5 | 31.03 |
| 75 | 0 | 196.5 | 0 |
| 75 | 0.26 | 196.5 | 0.84 |
| 75 | 4.44 | 196.5 | 1.65 |
| 75 | 7.42 | 196.5 | 2.92 |
| 75 | 10.34 | 196.5 | 3.95 |
| 75 | 13.62 | 196.5 | 5.02 |
| 75 | 17.26 | 196.5 | 6.09 |
| 75 | 21.27 | 196.5 | 7.12 |
| 75 | 25.64 | 196.5 | 8.16 |
| 75 | 30.38 | 196.5 | 9.16 |
| 75 | 35.84 | 196.5 | 10.22 |
| 75 | 42.03 | 196.5 | 11.26 |
| 75 | 48.96 | 196.5 | 12.28 |
| 75 | 56.61 | 196.5 | 13.28 |
| 75 | 64.62 | 196.5 | 14.21 |
| 75 | 72.64 | 196.5 | 14.99 |
| 75 | 80.65 | 196.5 | 15.71 |
| 75 | 88.67 | 196.5 | 16.35 |
| 75 | 96.68 | 196.5 | 16.93 |
| 75 | 104.70 | 196.5 | 17.43 |
| 75 | 112.71 | 196.5 | 17.89 |
| 75 | 120.73 | 196.5 | 18.31 |
| 75 | 128.74 | 196.5 | 18.69 |
| 75 | 136.76 | 196.5 | 19.03 |
| 75 | 144.77 | 196.5 | 19.35 |
| 75 | 152.79 | 196.5 | 19.65 |
| 75 | 160.80 | 196.5 | 19.92 |
| 75 | 168.82 | 196.5 | 20.17 |
| 75 | 176.83 | 196.5 | 20.40 |
| 75 | 184.85 | 196.5 | 20.61 |
| 75 | 192.86 | 196.5 | 20.85 |
| 75 | 200.87 | 196.5 | 21.10 |
| 75 | 208.89 | 196.5 | 21.28 |
| 75 | 216.90 | 196.5 | 21.39 |
| 75 | 224.92 | 196.5 | 21.56 |
| 75 | 232.93 | 196.5 | 21.72 |
| 75 | 240.95 | 196.5 | 21.88 |
| 75 | 248.96 | 196.5 | 22.10 |
| 75 | 256.98 | 196.5 | 22.19 |
| 75 | 264.99 | 196.5 | 22.25 |
| 75 | 273.01 | 196.5 | 22.38 |
| 75 | 281.02 | 196.5 | 22.48 |
| 75 | 289.04 | 196.5 | 22.58 |
| 75 | 297.05 | 196.5 | 22.68 |
| 75 | 305.07 | 196.5 | 22.78 |
| 75 | 313.08 | 196.5 | 22.88 |
| 75 | 321.10 | 196.5 | 22.96 |
| 75 | 329.11 | 196.5 | 23.03 |
| 75 | 337.13 | 196.5 | 23.13 |
| 75 | 345.14 | 196.5 | 23.20 |
| 75 | 353.16 | 196.5 | 23.28 |
| 75 | 361.17 | 196.5 | 23.33 |
| 75 | 369.19 | 196.5 | 23.32 |
| 75 | 377.20 | 196.5 | 23.45 |
| 75 | 385.22 | 196.5 | 23.54 |
| 75 | 393.23 | 196.5 | 23.60 |
| 75 | 401.25 | 196.5 | 23.67 |
| 75 | 409.26 | 196.5 | 23.71 |
| 75 | 417.28 | 196.5 | 23.78 |
| 75 | 425.29 | 196.5 | 23.85 |
| 75 | 433.31 | 196.5 | 23.89 |
| 75 | 441.32 | 196.5 | 23.96 |
| 75 | 449.34 | 196.5 | 24.00 |
| 75 | 453.71 | 196.5 | 24.00 |
| 75 | 328.5 | 0 | 0 |
| 75 | 328.5 | 0.29 | 0.47 |
| 75 | 328.5 | 2.89 | 1.42 |
| 75 | 328.5 | 5.17 | 2.59 |
| 75 | 328.5 | 7.64 | 3.75 |
| 75 | 328.5 | 10.31 | 4.90 |
| 75 | 328.5 | 13.16 | 6.02 |
| 75 | 328.5 | 16.40 | 7.16 |
| 75 | 328.5 | 20.01 | 8.32 |
| 75 | 328.5 | 24.01 | 9.49 |
| 75 | 328.5 | 28.19 | 10.57 |
| 75 | 328.5 | 32.38 | 11.55 |
| 75 | 328.5 | 36.57 | 12.44 |
| 75 | 328.5 | 40.75 | 13.26 |
| 75 | 328.5 | 44.94 | 14.02 |
| 75 | 328.5 | 49.12 | 14.71 |
| 75 | 328.5 | 53.31 | 15.34 |
| 75 | 328.5 | 57.50 | 15.93 |
| 75 | 328.5 | 61.68 | 16.47 |
| 75 | 328.5 | 65.87 | 16.97 |
| 75 | 328.5 | 70.06 | 17.46 |
| 75 | 328.5 | 74.24 | 17.83 |
| 75 | 328.5 | 78.46 | 18.28 |
| 75 | 328.5 | 82.61 | 18.68 |
| 75 | 328.5 | 86.80 | 19.04 |
| 75 | 328.5 | 90.99 | 19.38 |
| 75 | 328.5 | 95.17 | 19.68 |
| 75 | 328.5 | 99.36 | 19.98 |
| 75 | 328.5 | 103.54 | 20.27 |
| 75 | 328.5 | 107.73 | 20.53 |
| 75 | 328.5 | 111.92 | 20.81 |
| 75 | 328.5 | 116.10 | 21.11 |
| 75 | 328.5 | 120.29 | 21.26 |
| 75 | 328.5 | 124.48 | 21.52 |
| 75 | 328.5 | 128.66 | 21.72 |
| 75 | 328.5 | 132.85 | 22.00 |
| 75 | 328.5 | 137.03 | 22.14 |
| 75 | 328.5 | 141.22 | 22.30 |
| 75 | 328.5 | 145.41 | 22.48 |
| 75 | 328.5 | 149.59 | 22.65 |
| 75 | 328.5 | 153.78 | 22.80 |
| 75 | 328.5 | 157.96 | 22.97 |
| 75 | 328.5 | 162.15 | 23.13 |
| 75 | 328.5 | 166.34 | 23.27 |
| 75 | 328.5 | 170.52 | 23.41 |
| 75 | 328.5 | 174.71 | 23.54 |
| 75 | 328.5 | 178.90 | 23.68 |
| 75 | 328.5 | 183.08 | 23.81 |
| 75 | 328.5 | 187.27 | 23.91 |
| 75 | 328.5 | 191.45 | 24.02 |
| 75 | 328.5 | 195.64 | 24.14 |
| 75 | 328.5 | 199.83 | 24.26 |
| 75 | 328.5 | 204.01 | 24.36 |
| 75 | 328.5 | 208.20 | 24.46 |
| 75 | 328.5 | 212.38 | 24.62 |
| 75 | 328.5 | 216.57 | 24.66 |
| 75 | 328.5 | 220.76 | 24.76 |
| 75 | 328.5 | 224.94 | 24.86 |
| 75 | 328.5 | 229.13 | 24.96 |
| 75 | 328.5 | 231.60 | 25.01 |

The complete fitting curves from Fig. 2 of Ref. [8] were digitized here to obtain the values shown in the table. For each dataset, only one enzyme was varied and the other two were kept constant.

# Table S3. Generated dataset of enzyme activity ratios and their predicted pathway flux (*J_pred_*) by COPASI model with the added adjustment term (with ⍺= 3.09*10^6^)

| PGAM (mU) | ENO (mU) | PPDK (mU) | *J_pred_* (nmol·min^-1^) |
| --- | --- | --- | --- |
| 0 | 328.5 | 196.5 | 0 |
| 4.5 | 328.5 | 196.5 | 1.64543 |
| 6 | 328.5 | 196.5 | 2.18995 |
| 7.5 | 328.5 | 196.5 | 2.7325 |
| 9 | 328.5 | 196.5 | 3.27309 |
| 12 | 328.5 | 196.5 | 4.34838 |
| 13.5 | 328.5 | 196.5 | 4.88309 |
| 15 | 328.5 | 196.5 | 5.41585 |
| 16.5 | 328.5 | 196.5 | 5.94666 |
| 18 | 328.5 | 196.5 | 6.47553 |
| 21 | 328.5 | 196.5 | 7.52745 |
| 22.5 | 328.5 | 196.5 | 8.05052 |
| 24 | 328.5 | 196.5 | 8.57166 |
| 25.5 | 328.5 | 196.5 | 9.09086 |
| 27 | 328.5 | 196.5 | 9.60816 |
| 30 | 328.5 | 196.5 | 10.637 |
| 31.5 | 328.5 | 196.5 | 11.1486 |
| 33 | 328.5 | 196.5 | 11.6582 |
| 34.5 | 328.5 | 196.5 | 12.166 |
| 36 | 328.5 | 196.5 | 12.6718 |
| 39 | 328.5 | 196.5 | 13.6779 |
| 40.5 | 328.5 | 196.5 | 14.1781 |
| 42 | 328.5 | 196.5 | 14.6764 |
| 43.5 | 328.5 | 196.5 | 15.1729 |
| 45 | 328.5 | 196.5 | 15.6674 |
| 48 | 328.5 | 196.5 | 16.651 |
| 49.5 | 328.5 | 196.5 | 17.14 |
| 51 | 328.5 | 196.5 | 17.6271 |
| 52.5 | 328.5 | 196.5 | 18.1124 |
| 54 | 328.5 | 196.5 | 18.5959 |
| 57 | 328.5 | 196.5 | 19.5573 |
| 58.5 | 328.5 | 196.5 | 20.0352 |
| 60 | 328.5 | 196.5 | 20.5114 |
| 61.5 | 328.5 | 196.5 | 20.9857 |
| 63 | 328.5 | 196.5 | 21.4582 |
| 66 | 328.5 | 196.5 | 22.3977 |
| 67.5 | 328.5 | 196.5 | 22.8647 |
| 69 | 328.5 | 196.5 | 23.33 |
| 70.5 | 328.5 | 196.5 | 23.7935 |
| 72 | 328.5 | 196.5 | 24.2551 |
| 75 | 328.5 | 196.5 | 25.1731 |
| 76.5 | 328.5 | 196.5 | 25.6295 |
| 78 | 328.5 | 196.5 | 26.084 |
| 79.5 | 328.5 | 196.5 | 26.5368 |
| 81 | 328.5 | 196.5 | 26.9878 |
| 84 | 328.5 | 196.5 | 27.8846 |
| 85.5 | 328.5 | 196.5 | 28.3304 |
| 87 | 328.5 | 196.5 | 28.7744 |
| 88.5 | 328.5 | 196.5 | 29.2167 |
| 90 | 328.5 | 196.5 | 29.6572 |
| 93 | 328.5 | 196.5 | 30.5332 |
| 94.5 | 328.5 | 196.5 | 30.9686 |
| 96 | 328.5 | 196.5 | 31.4022 |
| 97.5 | 328.5 | 196.5 | 31.8342 |
| 99 | 328.5 | 196.5 | 32.2644 |
| 102 | 328.5 | 196.5 | 33.1198 |
| 103.5 | 328.5 | 196.5 | 33.545 |
| 105 | 328.5 | 196.5 | 33.9685 |
| 106.5 | 328.5 | 196.5 | 34.3903 |
| 108 | 328.5 | 196.5 | 34.8104 |
| 75 | 0 | 196.5 | 0 |
| 75 | 10.5 | 196.5 | 2.82162 |
| 75 | 22.5 | 196.5 | 5.60461 |
| 75 | 30 | 196.5 | 7.13554 |
| 75 | 33 | 196.5 | 7.7076 |
| 75 | 40.5 | 196.5 | 9.04545 |
| 75 | 52.5 | 196.5 | 10.9403 |
| 75 | 55.5 | 196.5 | 11.3718 |
| 75 | 63 | 196.5 | 12.3845 |
| 75 | 67.5 | 196.5 | 12.9502 |
| 75 | 78 | 196.5 | 14.1609 |
| 75 | 85.5 | 196.5 | 14.9419 |
| 75 | 90 | 196.5 | 15.3805 |
| 75 | 97.5 | 196.5 | 16.0662 |
| 75 | 108 | 196.5 | 16.9406 |
| 75 | 112.5 | 196.5 | 17.2879 |
| 75 | 120 | 196.5 | 17.8339 |
| 75 | 123 | 196.5 | 18.0415 |
| 75 | 135 | 196.5 | 18.8167 |
| 75 | 142.5 | 196.5 | 19.2603 |
| 75 | 145.5 | 196.5 | 19.4298 |
| 75 | 153 | 196.5 | 19.8351 |
| 75 | 165 | 196.5 | 20.4338 |
| 75 | 168 | 196.5 | 20.5747 |
| 75 | 175.5 | 196.5 | 20.9129 |
| 75 | 180 | 196.5 | 21.1068 |
| 75 | 190.5 | 196.5 | 21.5348 |
| 75 | 198 | 196.5 | 21.8214 |
| 75 | 202.5 | 196.5 | 21.9864 |
| 75 | 210 | 196.5 | 22.2502 |
| 75 | 220.5 | 196.5 | 22.5983 |
| 75 | 225 | 196.5 | 22.7404 |
| 75 | 232.5 | 196.5 | 22.9684 |
| 75 | 235.5 | 196.5 | 23.0567 |
| 75 | 247.5 | 196.5 | 23.3944 |
| 75 | 255 | 196.5 | 23.5936 |
| 75 | 258 | 196.5 | 23.6709 |
| 75 | 265.5 | 196.5 | 23.8585 |
| 75 | 277.5 | 196.5 | 24.1431 |
| 75 | 280.5 | 196.5 | 24.2113 |
| 75 | 288 | 196.5 | 24.3774 |
| 75 | 292.5 | 196.5 | 24.474 |
| 75 | 303 | 196.5 | 24.6909 |
| 75 | 310.5 | 196.5 | 24.839 |
| 75 | 315 | 196.5 | 24.9252 |
| 75 | 322.5 | 196.5 | 25.0649 |
| 75 | 333 | 196.5 | 25.2523 |
| 75 | 337.5 | 196.5 | 25.3299 |
| 75 | 345 | 196.5 | 25.4557 |
| 75 | 348 | 196.5 | 25.5048 |
| 75 | 360 | 196.5 | 25.6949 |
| 75 | 367.5 | 196.5 | 25.8088 |
| 75 | 370.5 | 196.5 | 25.8533 |
| 75 | 378 | 196.5 | 25.9622 |
| 75 | 390 | 196.5 | 26.1296 |
| 75 | 393 | 196.5 | 26.1702 |
| 75 | 400.5 | 196.5 | 26.2695 |
| 75 | 405 | 196.5 | 26.3276 |
| 75 | 415.5 | 196.5 | 26.4594 |
| 75 | 423 | 196.5 | 26.5504 |
| 75 | 427.5 | 196.5 | 26.6037 |
| 75 | 435 | 196.5 | 26.6906 |
| 75 | 445.5 | 196.5 | 26.8082 |
| 75 | 450 | 196.5 | 26.8573 |
| 75 | 328.5 | 0 | 0 |
| 75 | 328.5 | 4.5 | 1.65114 |
| 75 | 328.5 | 9 | 3.23942 |
| 75 | 328.5 | 13.5 | 4.76383 |
| 75 | 328.5 | 18 | 6.22352 |
| 75 | 328.5 | 22.5 | 7.61782 |
| 75 | 328.5 | 27 | 8.94626 |
| 75 | 328.5 | 31.5 | 10.2086 |
| 75 | 328.5 | 36 | 11.4049 |
| 75 | 328.5 | 40.5 | 12.5352 |
| 75 | 328.5 | 45 | 13.6002 |
| 75 | 328.5 | 49.5 | 14.6005 |
| 75 | 328.5 | 54 | 15.5372 |
| 75 | 328.5 | 58.5 | 16.4115 |
| 75 | 328.5 | 63 | 17.2249 |
| 75 | 328.5 | 67.5 | 17.9792 |
| 75 | 328.5 | 72 | 18.6763 |
| 75 | 328.5 | 76.5 | 19.3186 |
| 75 | 328.5 | 81 | 19.9084 |
| 75 | 328.5 | 85.5 | 20.4481 |
| 75 | 328.5 | 90 | 20.9406 |
| 75 | 328.5 | 94.5 | 21.3887 |
| 75 | 328.5 | 99 | 21.7951 |
| 75 | 328.5 | 103.5 | 22.1628 |
| 75 | 328.5 | 108 | 22.4948 |
| 75 | 328.5 | 112.5 | 22.7939 |
| 75 | 328.5 | 117 | 23.0629 |
| 75 | 328.5 | 121.5 | 23.3047 |
| 75 | 328.5 | 126 | 23.5217 |
| 75 | 328.5 | 130.5 | 23.7167 |
| 75 | 328.5 | 135 | 23.8917 |
| 75 | 328.5 | 139.5 | 24.0493 |
| 75 | 328.5 | 144 | 24.1912 |
| 75 | 328.5 | 148.5 | 24.3194 |
| 75 | 328.5 | 153 | 24.4356 |
| 75 | 328.5 | 157.5 | 24.5412 |
| 75 | 328.5 | 162 | 24.6376 |
| 75 | 328.5 | 166.5 | 24.726 |
| 75 | 328.5 | 171 | 24.8073 |
| 75 | 328.5 | 175.5 | 24.8826 |
| 75 | 328.5 | 180 | 24.9526 |
| 75 | 328.5 | 184.5 | 25.0179 |
| 75 | 328.5 | 189 | 25.079 |
| 75 | 328.5 | 193.5 | 25.1366 |
| 75 | 328.5 | 198 | 25.1574 |
| 75 | 328.5 | 202.5 | 25.1118 |
| 75 | 328.5 | 207 | 25.0684 |
| 75 | 328.5 | 211.5 | 25.0269 |
| 75 | 328.5 | 216 | 24.9873 |
| 75 | 328.5 | 220.5 | 24.9494 |
| 75 | 328.5 | 225 | 24.913 |
| 75 | 328.5 | 229.5 | 24.8781 |
| 75 | 328.5 | 232.5 | 24.8556 |
| 75 | 328.5 | 234 | 24.8446 |
| 75 | 328.5 | 237 | 24.8229 |
| 75 | 328.5 | 238.5 | 24.8123 |
| 75 | 328.5 | 241.5 | 24.7915 |
| 75 | 328.5 | 243 | 24.7813 |
| 75 | 328.5 | 246 | 24.7612 |
| 75 | 328.5 | 247.5 | 24.7513 |

For each dataset, only one enzyme activity was varied and the other two were kept constant.

# Table S4. Kinetic parameters of COPASI model using UUBB equation after optimization

| **Constant** | **Value (in µM)** |
| --- | --- |
| ***K_i_PEP_*** | 9 933 660 |
| ***K_i_AMP_*** | 2224.9 |
| ***K_i_PPi_*** | 9 948 210 |
| ***K_PPi_AMP_*** | 9 999 870 |
| ***K_ii_PPi_*** | 9 999 100 |

# Table S5. Flux control coefficient determination for models at physiological metabolite concentrations

| **Model** | **PGAM** | **ENO** | **PPDK** |
| --- | --- | --- | --- |
|  |  |  |  |
| **Moreno-Sanchez model [8]** | 0.77 | 0.18 | 0.05 |
| **Adjusted Moreno-Sanchez model** | 0.77 | 0.18 | 0.05 |
| **UUBB model** | 0.92 | 0.58 | 9.55*****10^-3^ |
| **UUBB model optimized** | 0.92 | 0.57 | 5.36*****10^-3^ |
| **Model with the added adjustment term (⍺= 3.09*10^6^)** | 0.77 | 0.18 | 0.05 |


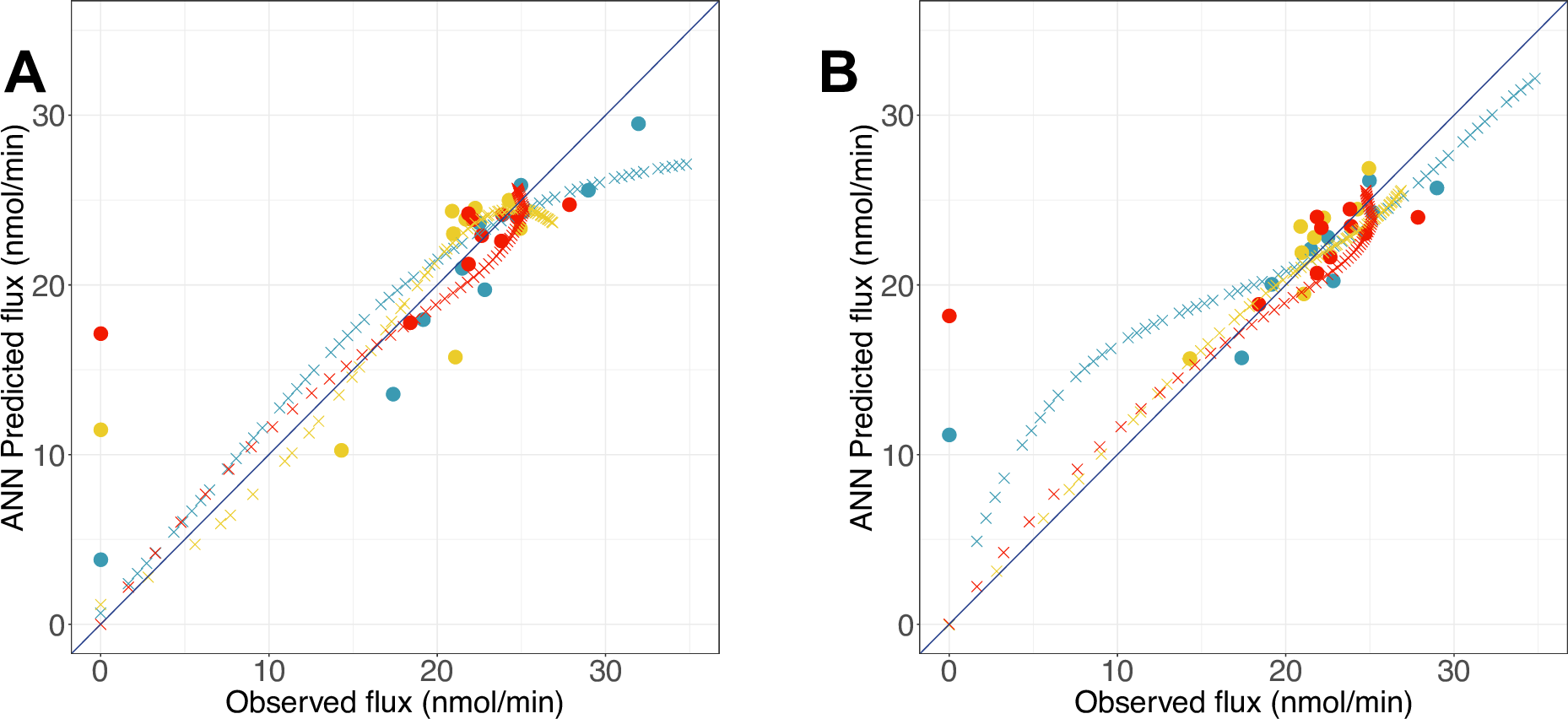


# **Fig S1. Flux predicted** by ANN models with the first and third dataset. (A) Flux prediction with NeuralNet and tanh activation function (4 HUs) with the first dataset. Train: cvRMSE= 4.47 nmol·min^-1^, cvMAE= 2.84 nmol·min^-1^, cvR^2^= 0.68 and Test: RMSE=2.01 nmol·min^-1^, MAE= 1.52 nmol·min^-1^, R^2^= 0.95. (B) Flux predicted with Nnet (2 HUs) with the first dataset. Train: cvRMSE= 4.56 nmol·min^-1^, cvMAE= 2.66 nmol·min^-1^, cvR^2^= 0.67 and Test: RMSE=2.43 nmol·min^-1^, MAE= 1.76 nmol·min^-1^, R^2^= 0.94.

# Circle colors refer to the varied enzyme activity: PGAM (blue), ENO (yellow) or PPDK (red).


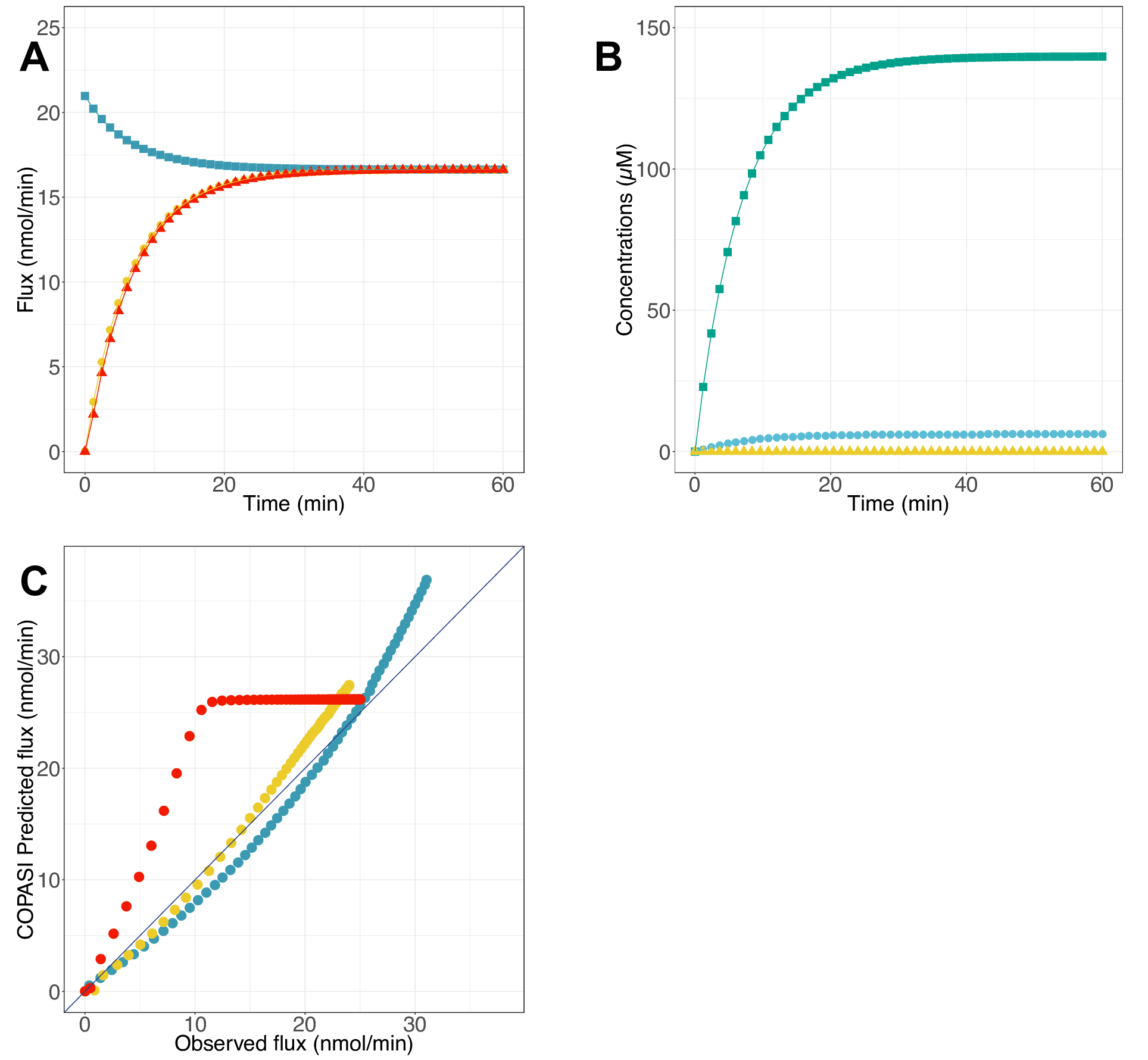


# Fig S2. Flux and metabolite concentration predictions with the Moreno-Sanchez model [8] using COPASI software. (A) PGAM (blue squares), ENO (yellow circles) and PPDK (red triangles) fluxes predicted as function of time. (B) Predicted concentrations of 2PG (green), PEP (blue) and Pyr (yellow). (C) Flux predicted by the model. RMSE= 4.33 nmol·min^-1^, MAE= 3.17 nmol·min^-1^, R^2^= 0.85. Circle colors refer to the varied enzyme activity: PGAM (blue), ENO (yellow) or PPDK (red).


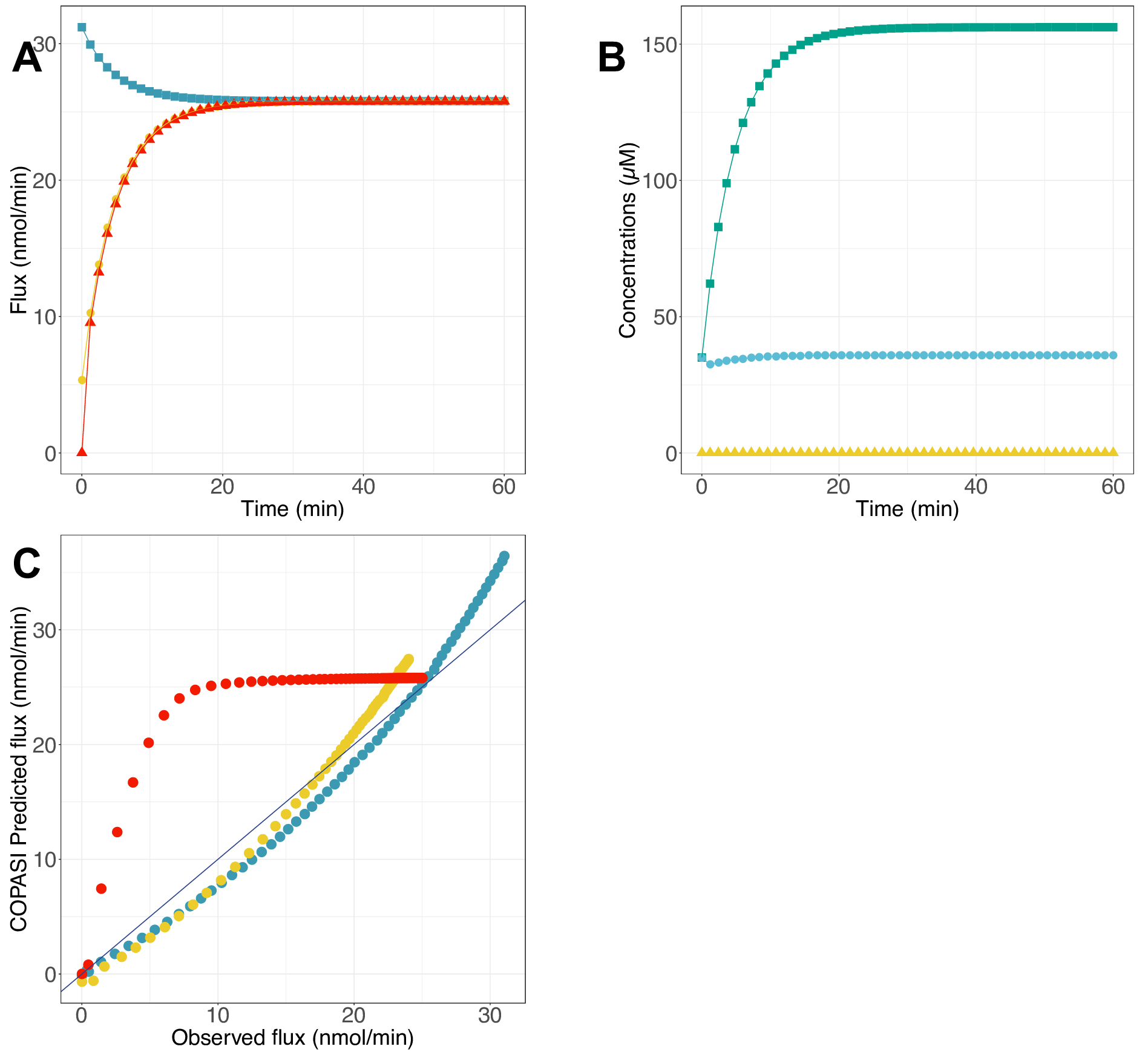


# Fig S3. Flux and metabolite concentration predictions with the lin-log approximation kinetics using COPASI software. Kinetic parameters used are from Table 1 and kinetic equations for PGAM and ENO from Table 2. Lin-log rate equation used for PPDK:

# $\boldsymbol{v=}\boldsymbol{V}_{\boldsymbol{max}}\boldsymbol{\cdot(1+}\boldsymbol{\varepsilon}_{\boldsymbol{A}}\boldsymbol{\cdot ln}\left( \frac{\left[ \boldsymbol{A} \right]}{\left[ \boldsymbol{A} \right]_{\boldsymbol{ss}}} \right)\boldsymbol{+}\boldsymbol{\varepsilon}_{\boldsymbol{P}}\boldsymbol{\cdot ln}\left( \frac{\left[ \boldsymbol{P} \right]}{\left[ \boldsymbol{P} \right]_{\boldsymbol{ss}}} \right)\boldsymbol{+}\boldsymbol{\varepsilon}_{\boldsymbol{I}}\boldsymbol{\cdot ln}\left( \frac{\boldsymbol{[I]}}{\boldsymbol{[I]}_{\boldsymbol{ss}}} \right)$),

with $V_{max}$, the maximum rates of each reaction;$\varepsilon_{A}$,$\varepsilon_{P}$ and $\varepsilon_{I}$ the elasticities for substrate (A), product (P) and inhibitor (I) and $\left[ \boldsymbol{A} \right]_{\boldsymbol{ss}}$,$\left[ \boldsymbol{P} \right]_{\boldsymbol{ss}}$ and ${\boldsymbol{[}\boldsymbol{I}\boldsymbol{]}}_{\boldsymbol{ss}}$ the steady-state concentrations of substrate, product and inhibitor. Concerning the parameters used here: $V_{max}$ are from Table 1, elasticities ($\varepsilon_{i}$) are estimated with Model 6 and steady-state concentrations are taken from Model 6.

# (A) PGAM (blue squares), ENO (yellow circles) and PPDK (red triangles) fluxes predicted as function of time. (B) Predicted concentrations of 2PG (green), PEP (blue) and Pyr (yellow). (C) Flux predicted by the model. RMSE= 4.8 nmol·min^-1^, MAE= 3.3 nmol·min^-1^, R^2^= 0.78. Circle colors refer to the varied enzyme activity: PGAM (blue), ENO (yellow) or PPDK (red).


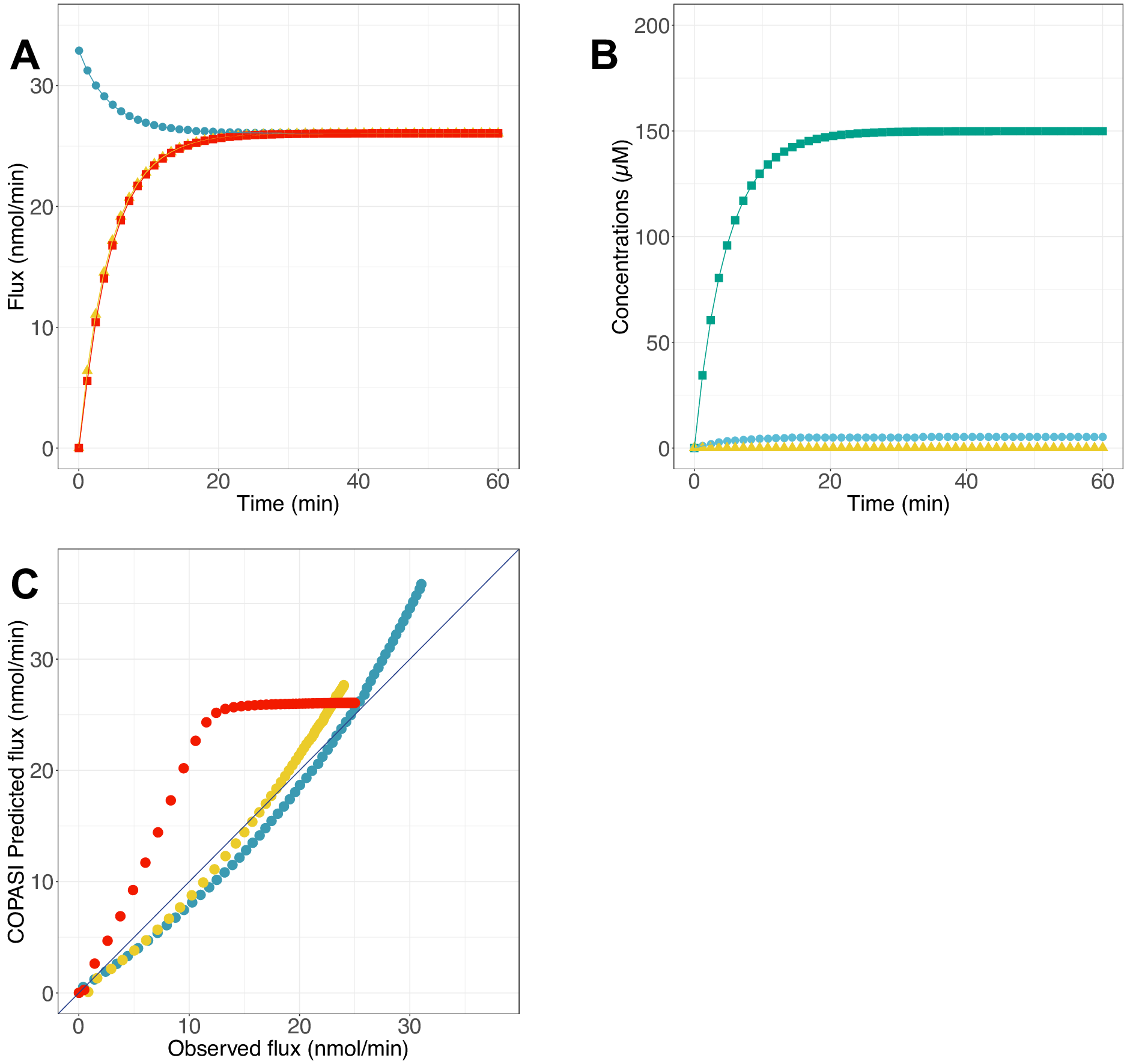


# Fig S4. Flux and metabolite concentration predictions with the modular rate law from Liebermeister using COPASI software. Kinetic parameters used are from Table 1 and kinetic equations for PGAM and ENO from Table 2. Modular rate law equation used for PPDK:

#

$$\boldsymbol{v=}\frac{\boldsymbol{V}_{\boldsymbol{f}}\boldsymbol{\cdot}\frac{\boldsymbol{PEP}}{\boldsymbol{K}_{\boldsymbol{mPEP}}}\boldsymbol{\cdot}\frac{\boldsymbol{AMP}}{\boldsymbol{K}_{\boldsymbol{mAMP}}}\boldsymbol{\cdot}\frac{\boldsymbol{PPi}}{\boldsymbol{K}_{\boldsymbol{mPPi}}}\boldsymbol{-}\boldsymbol{V}_{\boldsymbol{r}}\boldsymbol{\cdot}\frac{\boldsymbol{Pyr}}{\boldsymbol{K}_{\boldsymbol{mPyr}}}\boldsymbol{\cdot}\frac{\boldsymbol{ATP}}{\boldsymbol{K}_{\boldsymbol{mATP}}}\boldsymbol{\cdot}\frac{\boldsymbol{Pi}}{\boldsymbol{K}_{\boldsymbol{mPi}}}}{\left( \boldsymbol{1+}\frac{\boldsymbol{PEP}}{\boldsymbol{K}_{\boldsymbol{mPEP}}} \right)\boldsymbol{\cdot}\left( \boldsymbol{1+}\frac{\boldsymbol{AMP}}{\boldsymbol{K}_{\boldsymbol{m}\boldsymbol{AMP}}} \right)\boldsymbol{\cdot}\left( \boldsymbol{1+}\frac{\boldsymbol{PPi}}{\boldsymbol{K}_{\boldsymbol{mPPi}}} \right)\boldsymbol{+}\left( \boldsymbol{1+}\frac{\boldsymbol{Pyr}}{\boldsymbol{K}_{\boldsymbol{mPyr}}} \right)\boldsymbol{\cdot}\left( \boldsymbol{1+}\frac{\boldsymbol{ATP}}{\boldsymbol{K}_{\boldsymbol{mATP}}} \right)\boldsymbol{\cdot}\left( \boldsymbol{1+}\frac{\boldsymbol{Pi}}{\boldsymbol{K}_{\boldsymbol{mPi}}} \right)\boldsymbol{-1}}$$

# (A) PGAM (blue squares), ENO (yellow circles) and PPDK (red triangles) fluxes predicted as function of time. (B) Predicted concentrations of 2PG (green), PEP (blue) and Pyr (yellow). (C) Flux predicted by the model. RMSE= 4.03 nmol·min^-1^, MAE= 2.99 nmol·min^-1^, R^2^= 0.87. Circle colors refer to the varied enzyme activity: PGAM (blue), ENO (yellow) or PPDK (red). (D)


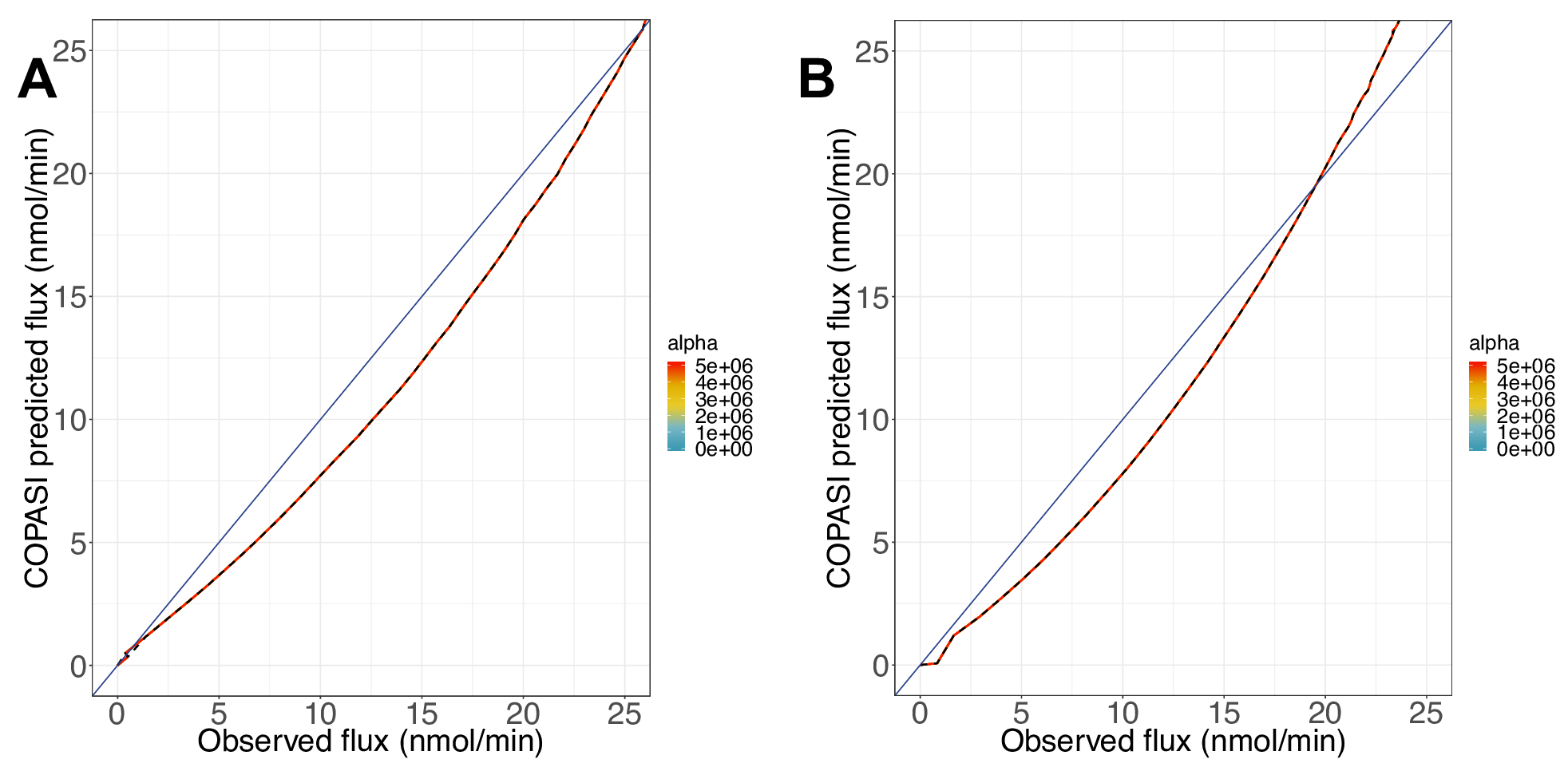


# Fig S5. Effect of the variation of$\boldsymbol{\alpha}$ in the adjustment term ($\boldsymbol{\alpha}\left| \boldsymbol{V}_{\boldsymbol{f}}\mathbf{-}\boldsymbol{V}_{\boldsymbol{f}\boldsymbol{0}} \right|$) of COPASI model. (A) Flux predictions by the model when PGAM activity is varied. (B) Flux predictions by the model when ENO activity is varied. Dotted line: curve obtained with the best adjustment term (3088970).
